# Multi-Anti-Parasitic Activity of Arylidene Ketones and Thiazolidene Hydrazines against *Trypanosoma cruzi* and *Leishmania* spp.

**DOI:** 10.3390/molecules22050709

**Published:** 2017-05-07

**Authors:** Guzmán Álvarez, Cintya Perdomo, Cathia Coronel, Elena Aguilera, Javier Varela, Gonzalo Aparicio, Flavio R. Zolessi, Nallely Cabrera, Celeste Vega, Miriam Rolón, Antonieta Rojas de Arias, Ruy Pérez-Montfort, Hugo Cerecetto, Mercedes González

**Affiliations:** 1 Laboratorio de Moléculas Bioactivas, CENUR Litoral Norte, Universidad de la República, Ruta 3 (km 363), Paysandú, C.P. 60000, Uruguay; cuquis266@gmail.com; 2 Centro Para el Desarrollo de la Investigación Científica (CEDIC/FMB/Diaz Gill Medicina Laboratorial), Asunción, C.P. 1255, Paraguay; cathiacoronel@gmail.com (C.C.); mcvegagomez@gmail.com (C.V.); rolonmiriam@gmail.com (M.R.); rojasdearias@gmail.com (A.R.d.A.); 3 Grupo de Química Medicinal-Laboratorio de Química Orgánica, Facultad de Ciencias, Universidad de la República, Montevideo, C.P. 11400, Uruguay; elepao168@gmail.com (E.A.); jvarelaubillos@gmail.com (J.V.); hcerecetto@gmail.com (H.C.); megonzal@fq.edu.uy (M.G.); 4 Sección Biología Celular, Facultad de Ciencias, Universidad de la República, Montevideo, C.P. 11400, Uruguay; gaparicio@fcien.edu.uy (G.A.); fzolessi@fcien.edu.uy (F.R.Z.); 5 Cell Biology of Neural Development Laboratory, Institut Pasteur de Montevideo, Montevideo, C.P. 11400, Uruguay; 6 Departamento de Bioquímica y Biología Estructural, Instituto de Fisiología Celular, Universidad Nacional Autónoma de México, Ciudad de México, C.P. 04510, Mexico; ncabrera@ifc.unam.mx (N.C.); ruy@ifc.unam.mx (R.P.-M.)

**Keywords:** anti-*T. cruzi* and anti-*Leishmania* spp. activity, arylidene ketones, thiazolidene hydrazines, triosephosphate isomerase, cruzipain, in vivo toxicity, zebrafish

## Abstract

A series of fifty arylideneketones and thiazolidenehydrazines was evaluated against *Leishmania infantum* and *Leishmania braziliensis*. Furthermore, new simplified thiazolidenehydrazine derivatives were evaluated against *Trypanosoma cruzi*. The cytotoxicity of the active compounds on non-infected fibroblasts or macrophages was established in vitro to evaluate the selectivity of their anti-parasitic effects. Seven thiazolidenehydrazine derivatives and ten arylideneketones had good activity against the three parasites. The IC_50_ values for *T. cruzi* and *Leishmania* spp. ranged from 90 nM–25 µM. Eight compounds had multi-trypanocidal activity against *T. cruzi* and *Leishmania* spp. (the etiological agents of cutaneous and visceral forms). The selectivity of these active compounds was better than the three reference drugs: benznidazole, glucantime and miltefosine. They also had low toxicity when tested in vivo on zebrafish. Trying to understand the mechanism of action of these compounds, two possible molecular targets were investigated: triosephosphate isomerase and cruzipain. We also used a molecular stripping approach to elucidate the minimal structural requirements for their anti-*T. cruzi* activity.

## 1. Introduction

Chagas disease and Leishmaniasis are neglected endemic protozoan diseases recognized as public health problems by the World Health Organization [1]. Chagas disease is found mainly in Latin America and is estimated to affect 6–7 million people, and at least 90–100 million inhabitants are exposed to risk in endemic areas; it is caused by the parasite *Trypanosoma cruzi* [2]. Important advances in vector control and transfusion of Chagas disease have been achieved in all of the Chagas Control Initiatives in the Americas; however, there is still a large number of chronic patients for whom treatment is not accessible or totally effective [3].

Leishmaniasis affects 12 million people in 98 countries, and a billion are exposed to risk (over 616 million for visceral and 413 million for cutaneous Leishmaniasis) [3]. Leishmaniasis is a group of diseases caused by more than 20 *Leishmania* species. There are four clinical forms of the disease: visceral Leishmaniasis (VL) (also called Kala-azar), post-kala-azar dermal Leishmaniasis (PKDL), cutaneous Leishmaniasis (CL) and mucocutaneous Leishmaniasis (MCL). However, the most common form is CL, and VL can be fatal if untreated [4].

An increase in cases of VL has been observed in the Southern Cone of Latin America, due to the great endemicity of canine reservoirs in urban and peri-urban areas [5,6]. A displacement to new areas free of VL cases has also been detected, like in Uruguay, where the first case of canine Leishmaniasis was reported in October 2015, with an initial confirmed canine prevalence of 18% in a few months [7].

It is important to note that no vaccines or drugs are available to prevent human infection with both diseases. The drugs for their treatments are limited, and the chemotherapeutic agents currently used are still inadequate due to their extreme toxicity [8]. Benznidazole (commercial names Radanil^®^ Abarax^®^ or Benznidazol^®^) and nifurtimox (Lampit^®^), currently used in Chagas disease treatment, both are almost 100% effective during the acute phase and in congenital cases, which represent between 5% and 10% of the cases; however, the efficacy of these drugs decreases during the chronic phase [2].

The drugs currently used for the treatment of CL and VL have quite serious adverse reactions. Teratogenic and cardiotoxic effects have been identified, as well as an increase in the resistance attributed to its use. It is therefore a priority to develop new, accessible and effective drugs [9,10]. For the treatment of LC and MLC, parenteral administration of pentavalent antimony (sodium stibogluconate or pentostam^®^ antimoniate and meglumine or glucantime^®^) is carried out for 20 days; and for VL, it is for 28 days. Both treatments are carried out under supervised medical control, greatly increasing their costs. An alarming resistance against these commercial drugs has been observed, while other drugs, such as pentamidine, rifampicin, amphotericin B, allopurinol and ketoconazole, also used to treat Leishmaniasis have limited therapeutic efficacy [9].

The etiological agents that cause both diseases are closely-related organisms that share similar essential proteins. Therefore, the present study aims to search for simple compounds that have a dual activity against both parasites. [11].

**Figure 1:**
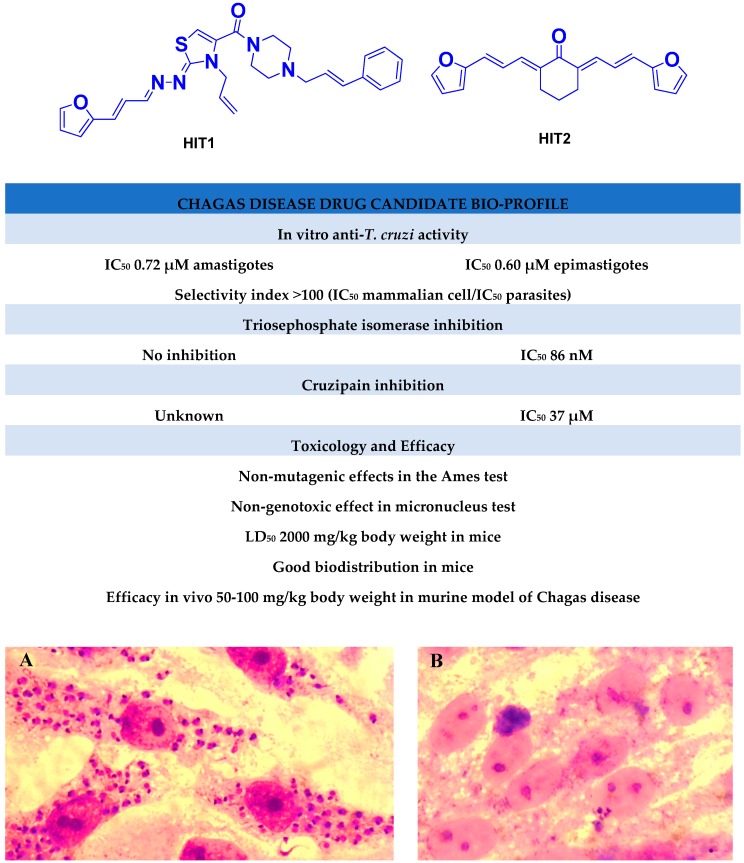
Drug candidates for Chagas disease, which also are leishmanicidal compounds. These compounds have an excellent profile as drugs for Chagas disease and are easy and inexpensive to produce. (**A**) A culture of Vero cells infected with *T. cruzi* (see the cytosol full of amastigotes); (**B**) The same cell culture after 72 h of treatment with 5 µM **HIT1** (see the cytosol without amastigotes) [12,13,19]

We have previously demonstrated the excellent trypanosomicidal activities of arylideneketones and thiadiazolidenehydrazines, together with their low toxicity and non-mutagenic properties (Figure 1) [12,13,14,15]. In these works, we identify two hits for the development of new anti-kinetoplastid compounds. **HIT1** has very good pharmacology profiles and was active in vivo in the acute model of Chagas disease. This compound was developed with a bioguided design of a hundred and fifty thiadiazolidenehydrazines using epimastigotes of *T. cruzi*. The activity of these molecules was then assayed in amastigotes, and a very good correlation between the activities in these two forms was observed. Furthermore, only the hydrosoluble members of this family lost the activity in the amastigote form. From these, **HIT1** was the best compound assayed in vivo, reducing the parasitemia by 70% at 100 mg/kg body weight in mice. In another approach, in a bioguided design using triosephosphate isomerase from *T. cruzi* (an essential glycolytic enzyme), we prepared around fifty arylideneketones and found the best inhibitor ever reported: **HIT2**. This series of compounds was designed thinking of the reported inhibitors of the triosephosphate isomerase, which are mostly symmetric molecules. Furthermore, we used in silico docking to design molecules directed to the interphase of this enzyme. The interphase is where the biggest structural differences are between the parasitic and the human enzyme, which could determine the selectivity of the new inhibitors. In addition, arylideneketones were recently reported as good antiparasitic agents. These compounds have antimalarial activity, and some are trypanosomicidal agents [16,17]. Moreover, the induction of oxidative stress was demonstrated for the arylideneketones in *T. cruzi*, *T. brucei* and *Leishmania* spp. [18] Following these criteria, we implemented a phenotypic screening of the active anti-*T. cruzi* molecules previously identified and tested their derivatives in vitro against *T. cruzi* and *Leishmania* spp., performing a molecular “strip-tease” to minimize their complexity, including their in vivo toxic activity on a zebrafish model.

## 2. Results and Discussion

### 2.1. Synthesis of **HIT1** and **HIT2** Derivatives

**Figure 2:**
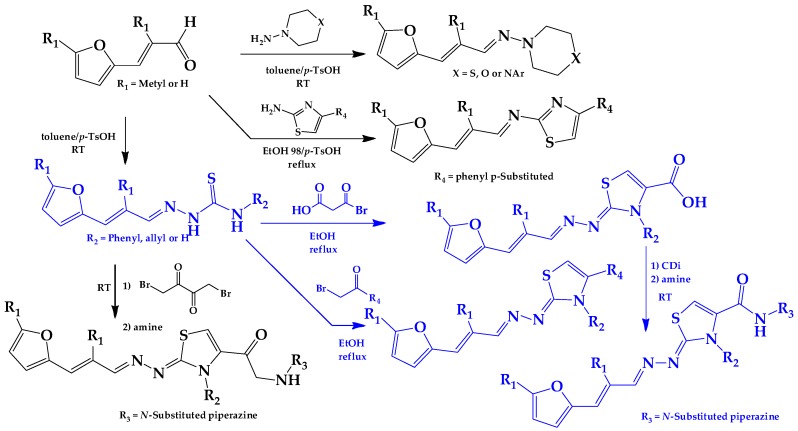
Synthetic procedures used to prepare the **HIT1** derivatives (blue) and for the molecular stripping (black). RT is room temperature 25 °C, CDi is 1,1′-carbonyldiimidazole.

Most of the compounds were synthetized previously as part of our ongoing program in drug development for Chagas disease [12,13,14,15]. For the synthesis of the thiazolidenehydrazine derivatives and for the molecular stripping (Figure 2), we used furylacroleine as the starting material. The aim of the molecular stripping was to elucidate the minimal structural requirement for the trypanocidal activity (see below).

**Figure 3:**
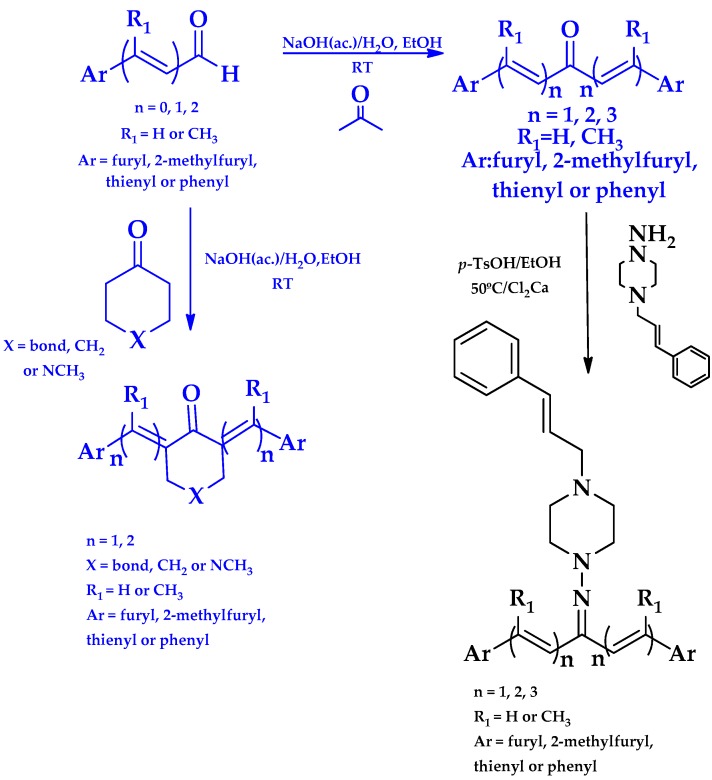
Synthetic procedures used to prepare the **HIT2** derivatives (blue) and for the molecular stripping (black).

We synthetized sixteen new compounds with good to excellent reaction yields. For the arylideneketone derivatives, we used a classical aldol reaction following the 12 green chemistry principles (Figure 3) [12]. All of the new compounds were characterized by ^1^H NMR, ^13^C NMR, COSY, HSQC and HMBC experiments and MS. The compounds, according to the H-H coupling constants and the NOE-diff experiments were obtained as the E-isomers. The purity of the synthesized compounds was established by TLC and microanalysis. Only compounds with analytical results for C, H, O, N and S, within ±0.4 of the theoretical values, were considered pure enough.

#### In Vitro Biological Studies

We initially selected compounds previously reported to have anti-*T. cruzi* activity from our chemical library [12,13,14,15] to evaluate their leishmanicidal activity. For this selection, we considered to be active all of the compounds with IC_50_ < 25 µM as an arbitrary cut off. To evaluate their activity in *Leishmania* spp., we performed a phenotypic screening using the promastigote form of *L. infantum* (MHOM/FR/91/LEM-2259) and *L. braziliensis* (MHOM/BR/75/M-2903) (Table 1A for the thiazolidenehydrazine derivate and Table 1B for the arylideneketones family). In order to evaluate the trypanocidal activity of the synthesized compounds in the molecular stripping approach, we used *T. cruzi*, Tulahuen 2 strain (genotype TcVI) [20] in the epimastigote form; initially working at 25 µM and when the percentage of growth inhibition was higher than 70%, we determined the IC_50_ (see Table S1 in the Supporting Information).

To establish the selectivity of the compounds against the parasites, nonspecific cytotoxicity against mammalian cells, using J774.1 murine macrophages and fibroblast NCTC929, was studied for the most relevant derivatives. The selectivity indexes were calculated as the ratio between cytotoxicity for mammalian cells and the anti-parasitic activity (Table 2).

We evaluated the anti-Kinetoplastid effect of forty compounds, previously active against *T. cruzi*, on *Leishmania* spp. These results are shown in Table 1A for the thiazolidenehydrazines family and Table 1B for the arylideneketones family. Fourteen compounds were found active against *Leishmania* spp. (IC_50_ < 25 µM), and eight of these compounds **GAT1033** (the compounds codes are exactly the same from the personal codes on the Lab, **GATN°**, **GATkN°**, **GATjmN°**, **EAN°** and **PgN°**), **GAT0113A**, **GAT1113**, **EA142**, **EA155**, **EA138**, **HIT1** and **HIT2**) had trypanocidal activity against the three species of parasites. The two hits, **HIT1** and **HIT2**, were active, but had lower activity and selectivity than the others. The best compound was **GAT1033** (Table 1A) with IC_50_ values of 1.6 µM, 7 µM and 2.0 µM for *T. cruzi*, *L. braziliensis* and *L. infantum*, respectively. The activity of **GAT1033** was better than that of benznidazole and glucantime. Compared with miltefosine, this compound has a better synthetic profile and also has an inexpensive and easier production. This compound could be prepared under green chemistry conditions. If we compare the leishmanicidal activities of compounds **GAT1033** (IC_50_ 2 µM), **GAT0113A** (IC_50_ 8 µM) and **GAT0513** (IC_50_ 58 µM), where the only structural change was the methyl group, we can see clear differences in the trypanocidal behaviors. On the one hand, derivative **GAT0513** (IC_50_ 90 nM) displayed improved trypanocidal activity on *T. cruzi*, being eighteen-times more active than **GAT1033**, but had a lower activity against *Leishmania* spp. If we compare the activities and selectivity of arylideneketones with previously reported compounds, i.e., curcumin [21] or **Comp13** (the compound code is exactly the same from the reference) [22], the values were better for the arylideneketones. **Comp13** is structurally very similar to our family of arylideneketones, but it did not display multi-anti-parasitic activity. When, in the series of arylideneketones, the cyclohexyl moiety of **HIT2** was changed by a methylpiperidinyl group, in derivative **Pg150** (see the Supporting Information file), to improve the solubility properties, the activity was completely lost.

Generally, the anti-parasitic activity was better against *T. cruzi* than against *Leishmania* spp., while *L. infantum* was more susceptible than *L. braziliensis* to the studied compounds. When compared, the thiazolidenehydrazine family was more active than the arylideneketones.

Eleven compounds showed good selectivity indexes (SI) 25-times more toxic than in the mammalian cells. Compounds **GAT1033**, **GAT0513**, **HIT1**, **EA155**, **EA141**, **EA160** and **EA156** displayed a better selectivity index than the reference drugs (SI ˃ 60); derivative **GAT1033** (SI = 200 vs. miltefosine SI = 56) being the most selective against *Leishmania* spp. and **GAT0513** (SI = 611 vs. benznidazole SI = 57) the most selective against *T. cruzi*.

All of these activities were shown in epimastigote and promastigote. As was previously reported by our group [12,13,14,15], there is a direct correlation between the activity in epimastigote and in amastigote for this kind of compounds, but there is no correlation with the activity against trypomastigotes.

### 2.2. In Vitro Molecular Stripping for *T. cruzi*

**Figure 4:**
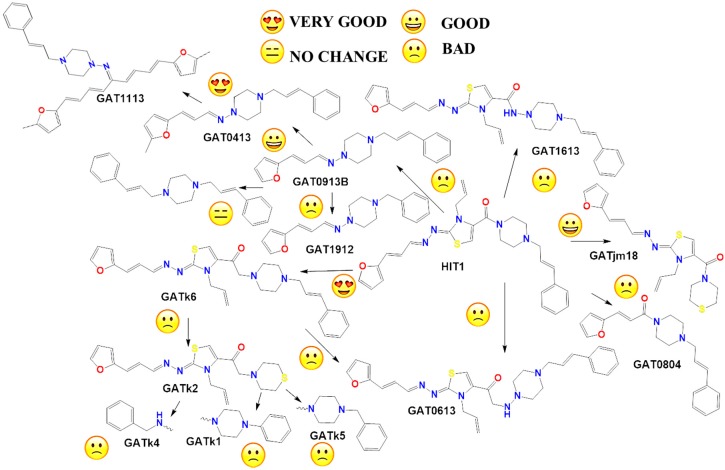
Molecular stripping of **HIT1** for *T. cruzi*. In the figure, we show, using the emoticon code, the different effects on the trypanocidal activity of our compounds caused by structural changes (very good (IC_50_ < 5 µM), good (IC_50_ 5–25 µM), no change and bad (IC_50_ ˃ 25 µM). The compounds codes are exactly the same from the personal codes on the Lab, **GATN°**, **GATkN°**, **GATjmN°**, **EAN°** and **PgN°**.

**Figure 5:**
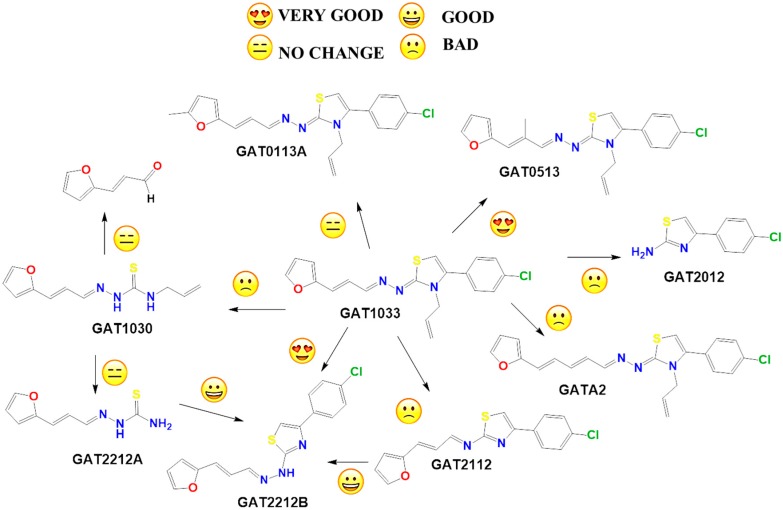
Molecular stripping of **GAT1033** for *T. cruzi*. In the figure, we show using the emoticon code the different effects on the trypanocidal activity of our compounds caused by structural changes (very good (IC_50_ < 5 µM), good (IC_50_ 5–25 µM), no change and bad (IC_50_ ˃ 25 µM).

We performed a procedure of molecular stripping for *T. cruzi* to elucidate the minimal structural requirements for a good trypanocidal effect (Figure 4 and Figure 5). The discussion is based on the structures of **HIT1** (Figure 4) and **GAT1033** (Figure 5) (for the complete data, see Supporting Information, Table S1). When we changed the trans-1-cinnamylpiperazinyl moiety, i.e., **GATjm18** (the compounds codes are exactly the same from the personal codes on the Lab, **GATN°**, **GATkN°**, **GATjmN°**, **EAN°** and **PgN°**), by a thiomorpholine motif, the activity was maintained. This motif is less expensive than the trans-1-cinnamylpiperazinyl motif, but in a biological milieu, could be susceptible to the oxidation of the sulfur atom. Another advantage of **GATjm18** is its lipophilicity and molecular weight, which fit the Lipinski rules. When the thiazole was omitted, i.e., in **GAT0913B**, the activity was lost; but, if we incorporated a methyl group, at the furanyl moiety (derivative **GAT0413**), the activity was restored. Remarkably, if we combine **EA155** with a cinnamylpiperazinyl motif, we obtain one of the most active compounds: **GAT1113**, with an IC_50_ of (1.6 ± 0.3) µM, two-times more active than **HIT1**. Furthermore, this compound is easier to synthesize than **HIT1** (with two steps less). In our previous work, we remarked that the furylacroleine motif is essential for trypanocidal activity; then, in **GAT1113**, we have this double motif [23] that explains the enhanced activity of this compound. Furthermore, this compound combines another pharmacophore: the cinnamylpiperazinyl motif. This could explain the eight-fold higher activity compared to **GAT0413**. The other highest scoring compound in this section was **GATk6** with an IC_50_ of (1.2 ± 0.3) µM. This compound was the result of changing the amide motif to a keto-amine motif. The aim of this change was to simplify the synthetic pathways that are involved in the activation of a carboxylic acid, where the use of toxic thionyl chloride or the expensive carbonyldiimidazole are clear disadvantages. Additionally, the reaction between the bromoketone intermediate and the amine has an excellent yield, compared to the 50% yield in the formation of the amide. In this way, when we changed the amide or amine for a hydrazide or a hydrazine moiety, respectively, the activity was lost (see derivatives **GAT1613** and **GAT0613**).

On the other hand, we performed the molecular stripping of **GAT1033**, which was the best compound with a multi-anti-parasitic effect (Figure 5). The absence of the furylacroleine motif, in derivative **GAT2012**, produces the lack of activity. The presence of a methyl group in different positions of the molecules was crucial to modulate the trypanocidal activity and, also, for the selectivity between parasites. For example, derivative **GAT0513** was active against *T. cruzi*, and derivative **GAT0113A** was also equipotent to the parent compound. The incorporation of additional insaturations between the furan ring and the hydrazine moiety did not improve the trypanocidal activity. If we took one of the nitrogens in the nitrogen triad, i.e., derivative **GAT2112**, the activity was lost, and when we had an unsubstituted nitrogen in the thiazol ring (which is aromatic), the activity was improved, i.e., derivative **GAT2212B** (IC_50_ = (1.2 ± 0.3) µM). The disadvantage of this compound is the number of tautomers and also of the isomers in the reaction. This was bad for the scaling process (mg–g), which gave low yields, quite different from the small-scale production (mg). The minimal structure could be **GAT2212B**, where the furylacroleine motif and the nitrogen triad are fixed. It would be interesting to incorporate an electron donor group, like a methyl moiety, in order to enhance the trypanocidal activity.

**Figure 6:**
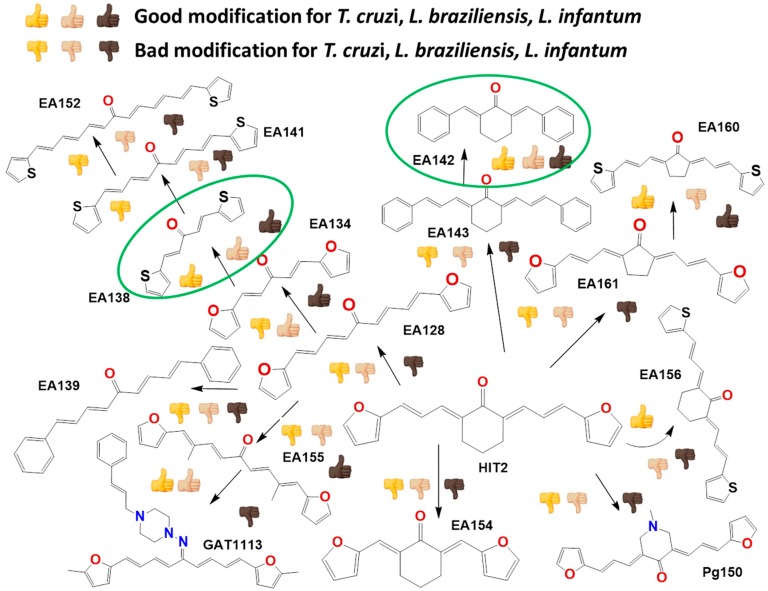
Multi-anti-parasitic activity comparison scheme. We show using the emoticon code a qualitative comparison for the trypanocidal activities of the arylideneketones on *T. cruzi*, *L. braziliensis* and *L. infantum* (good IC_50_ < 25 µM and bad IC_50_ ˃ 25 µM).

The compounds that had multi-anti-parasitic activities included diverse structures (Figure 6). Compounds **EA134** and **EA142** were two compounds with this type of activity. The triple anti-parasitic activity was lost when the number of insaturations increased. Furthermore, when the flexibility of the ketone was restricted by a cyclohexane, in **EA153**, this triple activity was lost, as compared to **EA134**. The incorporation and the positions of methyl groups, in this family of molecules, play an important role in the selectivity of the compounds between parasites (see **EA128** vs. **EA155**). Thus, **HIT2** looks like an optimized molecule; it cannot be modified. All of its modifications yield worse activity profiles. It is clear that the combination of active compounds like **EA138**, **EA155** and **HIT2** with cinnamylpiperazine could enhance trypanocidal activity (like in **GAT1113**).

### 2.3. Exploring the Mechanism of Action

In order to identify the potential modes of action of these agents, we made different kinds of experiments. Firstly, we evaluated the inhibition of the enzymatic activity of triosephosphate isomerase (TIM) (an essential glycolytic enzyme). The aim of this was to explore if previously described inhibitors of this enzyme from *T. cruzi* (*Tc*TIM) can also inhibit triosephosphate isomerase from *Leishmania* spp. (*Lm*TIM) [12]. Because these enzymes are very much conserved between organisms, they are constitutive enzymes. The structural similarity between TcTIM and *Lm*TIM is higher than with the human enzyme. No thiazolidenehydrazine inhibited the activity of TIM. Secondly, we also studied the inhibition of the activity of the protease cruzipain, another biological target relevant to the parasite. The aim of this study was explore if the piperazinyl motif, i.e., **HIT1**, previously described as a part of cruzipain inhibitors, was operative in our compounds, as a mode of action in *T. cruzi* [24]. Another mechanism of action concerning the oxidative stress induction was suggested for the arylideneketones, but we did not explore this possibility because these molecules have no nitro group in their structure.

#### 2.3.1. Inhibition of Triosephosphate Isomerase and Cruzipain

In order to investigate if the multi-trypanocidal agents derived from the phenotypic screening of *Leishmania* spp. inhibit the activity of triosephosphate isomerase from that parasite and if **HIT1** and other active derivatives inhibit cruzipain, we performed an initial screening of these molecules at a concentration of 100 µM. If some of these compounds inhibited more than 50% of the corresponding activity, we performed the inhibition assay at different concentrations of the compound to determine the IC_50_ values (Table 3). The inhibition profile of these compounds did not explain the total trypanocidal activity. We think another molecular target is involved in the case of *Leishmania* spp. because the TIM inhibition range is far from the leishmanicidal effect. Remarkably, inhibition of cruzipain activity was observed with **HIT1**; this compound has an amide group that acts as a mimetic substrate for this protease. It also has a piperazinyl motif, which has been described in other compounds as interacting closely with the active site of the enzyme [24]. **HIT1** has an IC_50_ of (4.3 ± 0.4) µM; this result is very good compared to recently-reported cruzipain inhibitors [25,26]. Furthermore, it is important to highlight that the assay was made with the pool of isoforms of cruzipain obtained directly from *T. cruzi*. This is important because the routine assay is usually made with cruzain (a recombinant enzyme), and that model is more sensitive to inhibition than cruzipain [27].

#### 2.3.2. Selective Inhibitors

We also performed activity inhibition studies with triosephosphate isomerase from *Homo sapiens* (*Hs*TIM), and these compounds did not affect this enzyme at 100 µM. From these results, we can say that the selectivity of these inhibitors is strong, because the inhibitor of *Tc*TIM does not inhibit *Lm*TIM and cruzipain and vice versa. Additionally, in order to know if these compounds could act as Michael acceptors, we performed a nucleophile challenge [28]. In this test, we incubated the inhibitors with cysteine, as a biological nucleophile, for 2 h under biological conditions. We did not observe any change in the chromatographic profile of the incubation medium. 

### 2.4. Toxicology In Vivo

For the exploration of the toxicological aspects of the best multi-antiparasitic agents, **HIT1** and **GAT1033**, we performed in vivo studies using zebrafish embryos.

#### 2.4.1. LD_50_ in Zebrafish Embryos and Developmental Toxicity 

**Figure 7:**
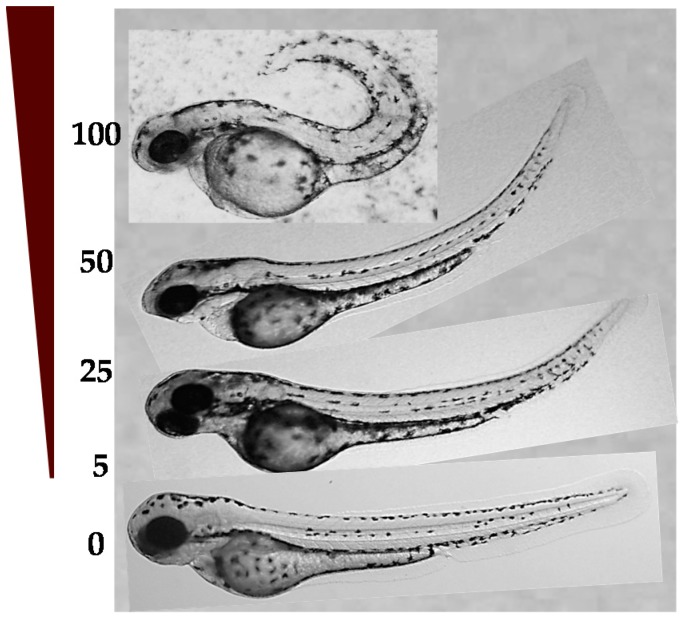
Dose-response effect of **GAT1033** on the curvature of the tail. Incubation concentrations were 0–300 µM for 72 h (illustrated with the brown triangle). The picture at the bottom shows a non-treated embryo at the same developmental stage.

We determined the lethal dose of six compounds, as detailed in Table S3 (see Supporting Information), using dechorionated zebrafish embryos at six hours post-fertilization (hpf). For this, the embryos were incubated with five different doses of the compounds for 72 h, and they were observed daily under a stereomicroscope and compared to normal control embryos (treated only with DMSO). In most cases, a dose-response effect was clearly observed. For example, in Figure 7, the effect of compound **GAT1033** at different doses in the curvature of the tail can be seen. The dose that kills 50% of the organisms (LD_50_) for compound **GAT1033** for embryos without chorion was 27 ± 1 µM and for **HIT1** 61 ± 2 µM. The toxicity was enhanced when the chorion was removed [29].

We found some limitations for testing lipophilic molecules like these in zebrafish embryos. For example, these compounds are insoluble in water, the medium in which embryos must be raised, and thus, the embryos themselves can be considered the only organic phase in this system. Hence, it would be expected that the compounds would be accumulated inside the embryos instead of the water. To test for this possibility, we performed some experiments to evaluate the concentration and distribution of the compounds inside the zebrafish embryos after incubation.

#### 2.4.2. Biodistribution of the Compound in a Zebrafish Model

**Figure 8:**
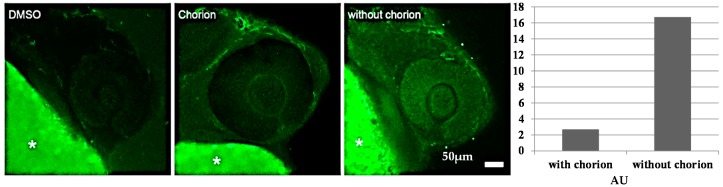
Differential distribution of **GAT1033** in embryos with and without chorion. The microscopy images on the left show the eye and parts of the yolk of 24-hpf zebrafish embryos. The intrinsic fluorescence of the compound was revealed using a laser scanning confocal microscope, after 18 h of incubation. The yolk (marked with *) shows a strong autofluorescence under these imaging conditions. The graph on the right shows the quantitation of fluorescence intensity (in arbitrary units AU) in treated embryos with and without chorion.

**Figure 9:**
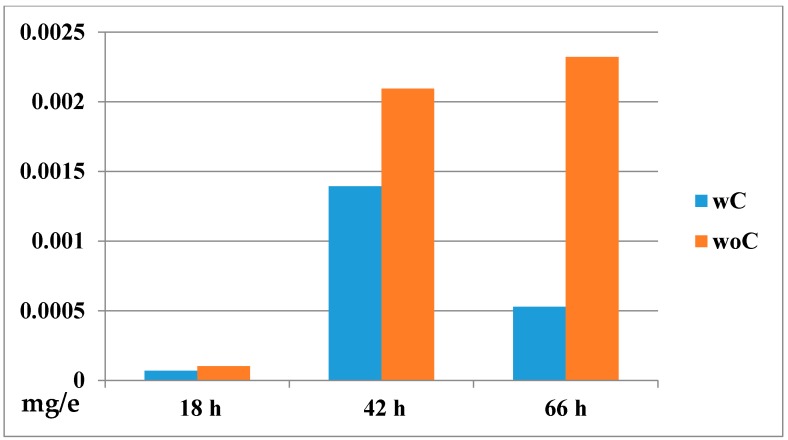
Bioconcentration effects of **GAT1033**. The bioconcentration effect can be seen at different incubation times. The embryos were incubated at 25 µM for up to 66 h. wC, with chorion; woC, without chorion. The graph shows the quantitation of the compound (in mg per embryo mg/e) in treated embryos with and without chorion.

To get an approximation of the distribution and concentration of **GAT1033** (the lipophilic compound) in the embryos, in the presence and absence of the chorion, we conducted a first analysis visualizing the embryos by laser scanning confocal microscopy. We observed significantly higher fluorescence intensity in embryonic tissues at 24 hpf (18 h incubation) in dechorionated embryos compared to embryos with chorion (Figure 8). We then quantified the internal concentration of **GAT1033** by UV-visible spectroscopy (Figure 9). To do this, we incubated embryos, with and without chorion, with a fixed dose of the compound for different extensions of time. After this, we performed a standard chemical extraction and quantified using spectrophotometry and checking the integrity of the molecules by thin layer chromatography.

Fish embryos are surrounded by a non-cellular cover generated during oogenesis, the chorion, which mostly provides them with mechanical protection, although it also acts as a relatively fine sieve, filtering out potential pathogens and even relatively large molecules [30]. Little attention has been given, however, to the possibility that some compounds tested in toxicity assays might have a limited diffusion capacity through this semipermeable membrane and might not reach the embryo at the desired concentrations. We observed that although the effects on the embryos of the most water-soluble molecule benznidazole (see Table S3 showing the solubility and lipophilic properties in the Supporting Information) appeared not to be affected by the presence of the chorion, there were significant differences in the effects of the compounds with reduced water solubility, when comparing embryos treated with and without the chorion. Nifurtimox showed a slightly different response, with a dose effect observed in the clumping of red blood cells near the heart, only in dechorionated embryos [29]. The potential toxic effects of the compound in the first 24 hpf, a time when very important developmental events occur, could be missed if it cannot pass through the chorion. Finally, we found that in many cases, the chorion of embryos treated with the test compounds lost their characteristic transparency, making it extremely difficult to observe the effects without removing it. Hence, even if the toxic effect could be observed at later stages, the effect on important morphogenic events, like brain and heart formation, might be missed if embryos are treated while inside the chorion. 

This could be explained by assuming that permeation of chemicals is reduced by the chorion as for organic pollutants and lipophilic drugs [31,32,33]. For that reason, it is necessary to take into account two parameters: bioaccumulation and chorion permeability [33]. Another important factor to take into consideration when designing and performing toxicity tests is the possibility that some compounds, particularly those that have a low water solubility, might be accumulated in embryo tissues or even the yolk, greatly affecting the pharmacokinetics of the drug [34]. The measurement of the internal drug concentration by spectroscopy indeed demonstrated that lipophilic compounds were being accumulated inside embryos, reaching concentrations a hundred-times higher than in the surrounding solution (Figure 9 and Table S4). The concentration of the compound in the embryo lysate was around 600 µM (using an estimation of the embryo volume), and the concentration in the incubation solution was 25 µM at 42 h of treatment; this is one hundred-times higher than expected. Interestingly, both with and without chorion, we observed that the concentration inside embryos was significantly higher than the expected by simple diffusion, where equilibrium would be reached between the medium and the embryo (around 25 µM). This accumulation defines the concomitant exacerbation of the toxic effect [35,36]. As most designed drugs are organic compounds, which tend to exhibit poor water solubility, we encourage researchers to include this type of analysis when performing toxicity tests, which would greatly increase accuracy in estimating the effect.

## 3. Experimental Section

### 3.1. General

Reagents were purchased from Aldrich and used without further purification. Melting points were performed using an Electrothermal Engineering Ltd. melting point apparatus, and the results were not corrected. ^1^H NMR and ^13^C NMR spectra were recorded in the indicated solvent with a Bruker DPX 400-MHz spectrometer. Chemical shifts are quoted in parts per million downfield from tetramethylsilane (TMS), and the coupling constants are in Hertz. Structural assignments were corroborated by HMBC and HSQC experiments. All solvents were dried and distilled prior to use. All of the reactions were carried out in a nitrogen atmosphere. Reactions were monitored by TLC using commercially available precoated plates (Merck Kieselgel 60 F254 silica), and the developed plates were examined under UV light (254 nm) or as iodine vapor stains. Column chromatography was performed using a 200 mesh silica gel. To determine the purity of the compounds, microanalyses were done on a Fisons EA 1108 CHNS–O instrument from vacuum-dried samples and were within ±0.4 of the values obtained by calculating their compositions. Compounds **GAT0804**, **GAT1030, GAT1033, HIT1, GATR22, GAT0921, GAT03311, GAT1048, GAT01811, GAT1050, GAT1066**, **GAT02111, GAT1049, GAT1057, GAT1058, GAT1075, GAT03211, GAT0812, GAT0212, GAT10117, GAT01911, GAT02211, GAT1082, EA155, EA161, EA134, EA128, HIT2, EA141, EA138, EA160, EA156, EA152, EA153** and **EA159** were prepared following synthetic procedures previously reported [12,15,16,17].

#### 3.1.1. General Synthetic-Procedure for Thiazolylidene Hydrazines **GAT0113A**, **GAT0513**, **GAT2015**, **GATA2** and **GAT2212b**

A mixture of the corresponding thiosemicarbazide [23] (1.0 eq.), the α-haloketone (1.2 eq.) and dry ethanol (1 mL per 100 mg of thiosemicarbazide) was heated at reflux until the disappearance of the thiosemicarbazide (4–10 h, checked by TLC, SiO_2_, petroleum ether:EtOAc 70:30). After that, the mixture was cooled at room temperature, and the precipitate was filtered off and washed with ethanol:water (80:20). The solid was crystallized from ethanol or ethanol:water.


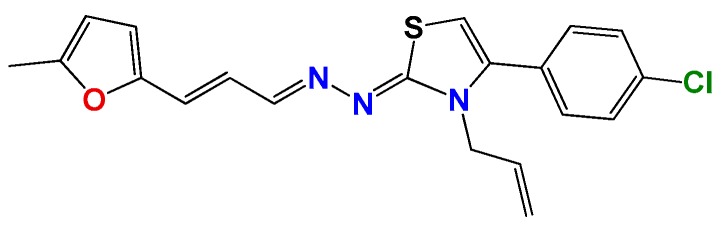


*1Z-[3-Allyl-4-(4-chlorophenyl)thiazol-2(3H)-ylidene]-2E-[3-(2methylfuryl)-2E-propenylidene hydrazine* (**GAT0113A**): Yield: 89%; yellow solid, m.p.: 220 °C (d); ^1^H NMR (400 MHz, CDCl_3_) δ: 7.50 (bs, 1H), 7.44 (d, *J* = 8 Hz, 2H), 7.32 (d, *J* = 8 Hz, 2H), 6.95 (s, 1H), 6.90 (d, *J* = 16 Hz, 1H), 6.85 (dd, *J* = 16, 6.0 Hz, 1H), 6.58 (d, *J* = 4 Hz, 1H), 6.08 (d, *J* = 4 Hz, 1H), 5.88 (d, *J* = 16 Hz, 1H), 5.25 (d, *J* = 10 Hz, 1H), 5.05 (d, *J* = 16 Hz, 1H), 4.71 (s, 2H), 2.30 (s, 3H); ^13^C NMR (100 MHz, CDCl_3_) δ: 168, 154, 152, 144, 140, 136, 131, 130, 130, 129, 126, 123, 117, 117, 112, 111, 103, 48, 14. MS (EI) *m*/*z* (abundance, %): 382.09 (M^+^, 100). Anal. calc. for C_20_H_18_ClN_3_OS: C, 62.57%; H, 4.73%; Cl, 9.24%; N, 10.95%; O, 4.17%; S, 8.35%; found: C, 62.6%; H, 4.7%; Cl, 9.3%; N, 11.0%; O, 4.2%; S, 8.4%.


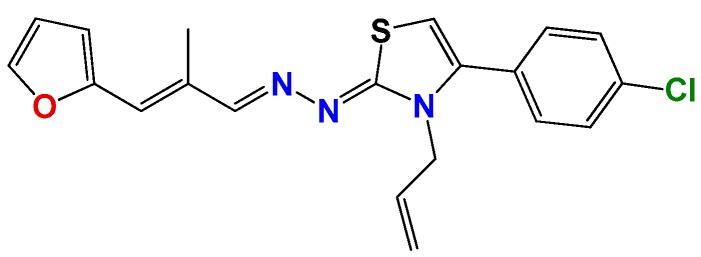


*1Z-[3-Allyl-4(4-chlorophenyl)thiazol-2(3H)-ylidene]-2E-[3-(2-furyl)-2-methyl-2E-propenylidene hydrazine* (**GAT0513**): Yield: 95%; yellow solid, m.p.: 228 °C (d); ^1^H NMR (400 MHz, CDCl_3_) δ: 7.75 (d, *J* = 6 Hz, 1H), 7.50 (bs, 1H), 7.44 (d, *J* = 8 Hz, 2H), 7.32 (d, *J* = 8 Hz, 2H), 6.95 (s, 1H), 6.90 (d, *J* = 16 Hz, 1H), 6.58 (d, *J* = 4 Hz, 1H), 6.08 (d, *J* = 4 Hz, 1H), 5.88 (d, *J* = 16 Hz, 1H), 5.25 (d, *J* = 10 Hz, 1H), 5.05 (d, *J* = 16 Hz, 1H), 4.71 (s, 2H), 2.21 (s, 3H); ^13^C NMR (100 MHz, CDCl_3_) δ: 168, 154, 152, 144, 140, 136, 131, 130, 130, 129, 126, 123, 117, 117, 112, 111, 103, 48, 14. MS (EI) *m*/*z* (abundance, %): 382.09 (M^+^, 100). Anal. calc. for C_20_H_18_ClN_3_OS: C, 62.57%; H, 4.73%; Cl, 9.24%; N, 10.95%; O, 4.17%; S, 8.35%; found: C, 62.6%; H, 4.7%; Cl, 9.3%; N, 11.0%; O, 4.2%; S, 8.4%.


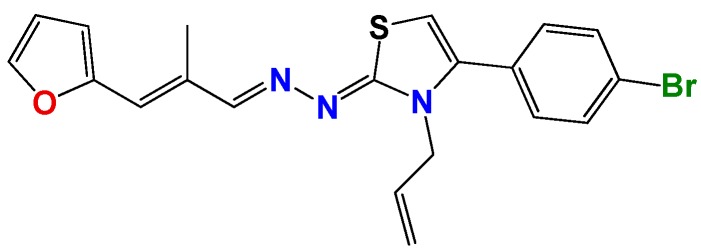


*1Z-[3-Allyl-4(4-bromophenyl)thiazol-2(3H)-ylidene]-2E-[3-(2-furyl)-2-methyl-2E-propenylidene hydrazine* (**GAT2015**): Yield: 98%; red solid, m.p.: 234 °C (d); ^1^H NMR (400 MHz, CDCl_3_) δ: 7.75 (d, *J* = 6 Hz, 1H), 7.50 (bs, 1H), 7.55 (d, *J* = 8 Hz, 2H), 7.27 (d, *J* = 8 Hz, 2H), 6.95 (s, 1H), 6.90 (d, *J* = 16 Hz, 1H), 6.58 (d, *J* = 4 Hz, 1H), 6.08 (d, *J* = 4 Hz, 1H), 5.88 (d, *J* = 16 Hz, 1H), 5.25 (d, *J* = 10 Hz, 1H), 5.05 (d, *J* = 16 Hz, 1H), 4.71 (s, 2H), 2.21 (s, 3H); ^13^C NMR (100 MHz, CDCl_3_) δ: 168, 154, 152, 144, 140, 136, 131, 130, 130, 129, 126, 123, 117, 117, 112, 111, 103, 48, 14. MS (EI) *m*/*z* (abundance, %): 426.04 (M^+^, 100). Anal. calc. for C_20_H_18_BrN_3_OS: C, 56.08%; H, 4.24%; Br, 18.65%; N, 9.81%; O, 3.74%; S, 7.49%; found: C, 56.1%; H, 4.3%; Br, 18.7%; N, 10.0%; O, 3.7%; S, 7.5%. 


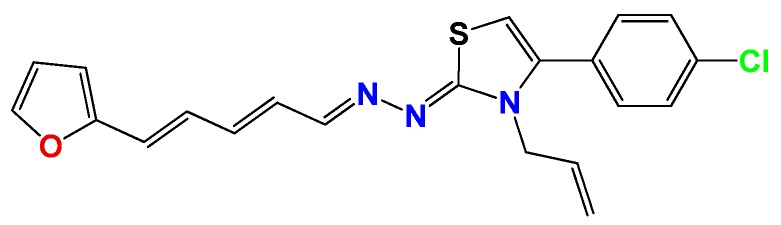


*1Z-[3-Allyl-4(4-chlorophenyl)thiazol-2(3H)-ylidene]-2E-[3-(2-furyl)-2E,4E-pentanylidene hydrazine* (**GATA2**): Yield: 58%; brown solid, m.p.: 134 °C (d); ^1^H NMR (400 MHz, CDCl_3_) δ: 7.75 (d, *J* = 6 Hz, 1H), 7.50 (bs, 1H), 7.55 (d, *J* = 8 Hz, 2H), 7.27 (d, *J* = 8 Hz, 2H), 6.95 (s, 1H), 6.71 (m, 1H), 6.65 (d, *J* = 16 Hz, 1H), 6.58 (d, *J* = 4 Hz, 1H), 6.55 (m, 1H), 6.08 (d, *J* = 4 Hz, 1H), 5.88 (d, *J* = 16 Hz, 1H), 5.25 (d, *J* = 10 Hz, 1H), 5.05 (d, *J* = 16 Hz, 1H), 4.71 (s, 2H); ^13^C NMR (100 MHz, CDCl_3_) δ: 168, 154, 152, 144, 140, 136, 131, 130, 130, 129, 127, 126, 123, 117, 117, 112, 111, 103, 48. MS (EI) *m*/*z* (abundance, %): 426.04 (M^+^, 100). Anal. calc. for C_20_H_18_BrN_3_OS: C, 56.08%; H, 4.24%; Br, 18.65%; N, 9.81%; O, 3.74%; S, 7.49%; found: C, 56.1%; H, 4.3%; Br, 18.7%; N, 10.0%; O, 3.7%; S, 7.5%. 


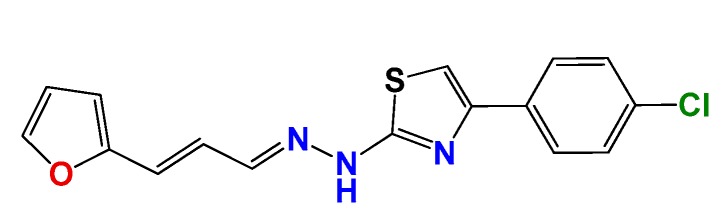


*1Z-[(4-chlorophenyl)thiazol-2(3H)-ylidene]-2E-[3-(2-furyl)-2E-propenylidene hydrazine* (**GAT2212b**): Yield: 89%; yellow solid, m.p.: 212 °C (d); ^1^H NMR (400 MHz, CDCl_3_) δ: 7.77 (s, 1H), 7.77 (d, *J* = 8 Hz, 2H), 7.75 (d, *J* = 6 Hz, 1H), 7.55(d, *J* = 8 Hz, 2H), 7.15 (d, *J* = 16 Hz, 1H), 6.95 (s, 1H), 6.85 (m, 2H), 6.42 (m, 2H), 6.19 (d, *J* = 16 Hz, 1H); ^13^C NMR (100 MHz, CDCl_3_) δ: 171, 151.5, 150, 144, 137, 134, 132, 131, 130, 129, 129, 128, 125, 114, 113, 105. MS (EI) *m*/*z* (abundance, %): 328.03 (M^+^, 100). Anal. calc. for C_16_H_12_ClN_3_OS: C, 58.27%; H, 3.67%; Cl, 10.75%; N, 12.74%; O, 4.85%; S, 9.72%; found: C, 58.3%; H, 3.7%; Cl, 10.7%; N, 12.7%; O, 4.9%; S, 9.7%.

#### 3.1.2. Synthetic Procedure for 4(4-Chlorophenyl)-thiazol-2(3*H*)-ylidene-2-amine (**GAT2012**)


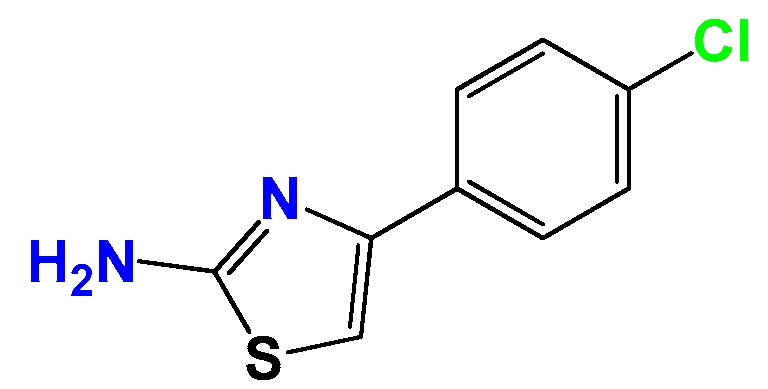


A mixture of thiourea (1.0 eq.), the α-haloketone (1.2 eq.) and dry ethanol (1 mL per 100 mg of thiourea) was heated at reflux until the disappearance of the thiourea (12 h, checked by TLC, SiO_2_, petroleum ether:EtOAc 70:30). After that, the mixture was cooled at room temperature, and the precipitated was filtered off and washed with ethanol:water (80:20). The solid was recrystallized from ethanol or ethanol:water. Yield: 98%; white solid, m.p. 167–168 °C; ^1^H NMR (400 MHz, CDCl_3_) δ: 7.74–7.70 (m, 2H), 7.38–7.33 (m, 2H), 6.73 (s, 1H), 5.07 (bs, 2H); ^13^C NMR (100 MHz, CDCl_3_) δ: 167.3, 150.2, 133.4, 133.1, 128.8, 127.3, 103.3. MS (EI) *m*/*z* (abundance, %): 211.01 (M^+^, 100). Anal. calc. for C_9_H_9_ClN_2_S: C, 51.06%; H, 3.81%; Cl, 16.75%; N, 13.23%; S, 15.15%; found: C, 51.0%; H, 3.8%; Cl, 16.8%; N, 13.2%; S, 15.2%.

#### 3.1.3. Synthetic Procedure of (*E*)-4-(4-Chlorophenyl)-*N*-((*E*)-3-(furan-2-yl)allylidene)thiazol-2-amine (**GAT2112**)


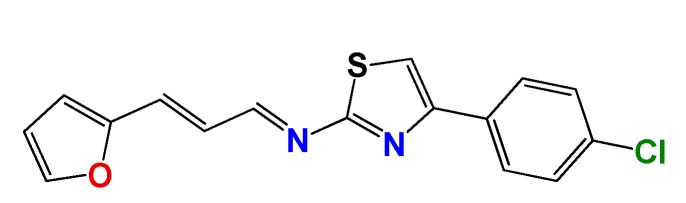


A mixture of furylacroleine (1.0 eq.), **GAT2012** (1.2 eq.) and dry ethanol (1 mL per 100 mg of **GAT2012**) with a catalytic amount of Et_3_N was heated at reflux until the disappearance of the aldehyde (12 h, checked by TLC, SiO_2_, petroleum ether:EtOAc 70:30). After that, the mixture was cooled to room temperature, and the precipitate was filtered off and washed with ethanol:water (80:20). The solid was crystallized from ethanol or ethanol:water. Yield: 68%; yellow solid, m.p. 185 °C (d); ^1^H NMR (400 MHz, CDCl_3_) δ: 8.82 (d, *J* = 9.3 Hz, 1H), 7.94–7.78 (m, 2H), 7.54 (d, *J* = 1.8 Hz, 1H), 7.42–7.40 (m, 2H), 7.38 (s, 1H), 7.15 (d, *J* = 16 Hz, 1H), 7.02 (dd, *J* = 16, 9.3 Hz, 1H), 6.70 (d, *J* = 3.4 Hz, 1H), 6.54 (dd, *J* = 3.4, 1.8 Hz, 1H); ^13^C NMR (100 MHz, CDCl_3_) δ: 173, 163, 151, 143, 134, 131, 129, 124, 114, 113, 111. MS (EI) *m*/*z* (abundance, %): 313.02 (M^+^, 100). Anal. calc. for C_16_H_11_ClN_2_OS: C, 61.05%; H, 3.52%; Cl, 11.26%; N, 8.90%; O, 5.08%; S, 10.19%; found: C, 61.1%; H, 3.5%; Cl, 11.3%; N, 8.9%; O, 5.1%; S, 10.2%.

#### 3.1.4. General Synthetic Procedure for Piperazine-Hydrazine Derivatives


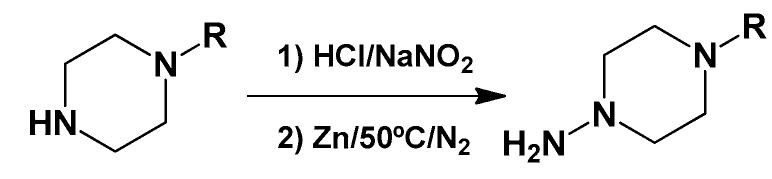


The corresponding amine (4.0 eq.) was added slowly to an aqueous solution of HCl 20%; the mixture was stirred for 2 h at room temperature. Then, an aqueous solution of-NaNO_2_ (4.0 eq.) was added slowly in an ice bath, and the mixture was stirred for 1 h. This solution was neutralized with NaOH and extracted with dichloromethane. The organic phase was dried with sodium sulfate, and the solvent was evaporated in vacuo. The resultant solid was dissolved in acetic acid and water over a N_2_ flux (1:1), and 16 eq. of Zn (dust) were added in four portions every 20 min. Then, the mixture was heated to 50 °C for 1 h. After the reaction was finished, the excess of Zn was hot filtered over zigzag folded paper. Then, this solution was neutralized with NaOH and extracted with dichloromethane. The organic phase was dried with sodium sulfate, and the solvent was evaporated. We directly used this mixture for the following reactions: 3.2.5 and 3.2.6.

#### 3.1.5. Synthetic Procedure for Thiazolylidene Hydrazides **GATk1**, **GATk2**, **GATk4**, **GATk5**, **GATk6** and **GAT0613**

A mixture of *1-((Z)-3-allyl-2-((E)-((E)-3-(furan-2-yl)allylidene)hydrazono)-2,3-dihydrothiazol-4-yl)-2-bromoethanone* [14] (1.0 eq.), the corresponding hydrazine or amine (1.2 eq.) and dry ethanol (1 mL per 100 mg of reagent) was heated at reflux until the disappearance of the reagents (4–10 h, checked by TLC, SiO_2_, petroleum ether:EtOAc 50:50). After that, the mixture was cooled to room temperature, and the precipitate was filtered off and washed with ethanol:water (80:20). The solid was crystallized from ethanol or ethanol:water.


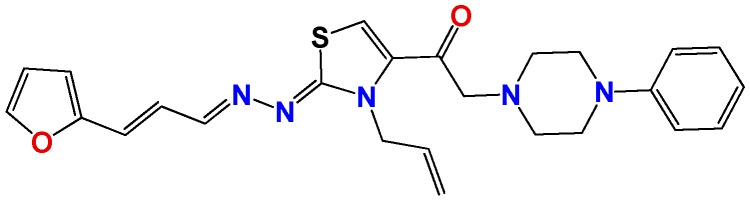


*(2E,2Z)-2-(2-((E)-3-(furan-2-yl)propeniliden)hydrazono)-(3-allyl-2,3-dihydrothiazol-4-yl)-2-(4-phenylpiperazin-1-yl)ethanone* (**GATk1**): Yield: 25% dark oil, ^1^H NMR (400 MHz, CDCl_3_) δ: 8.09 (m, 1H), 7.68 (s, 1H), 7.44 (bs, 1H), 7.31 (m, 2H), 6.97 (m 1H), 6.94 (m, 3H), 6.66 (d, *J* = 16 Hz, 1H), 6.43 (bs, 2H), 5.95 (m, 1H), 5.18 (m, 2H), 4.93 (bs, 2H), 3.76 (bs, 4H), 3.32 (bs, 2H), 2.87 (bs, 4H); ^13^C NMR (100 MHz, CDCl_3_) δ: 186, 166, 154, 153, 143, 132, 129, 126, 125, 124, 120, 117, 112, 64, 53, 48; MS (EI) *m*/*z* (abundance, %): 460.18 (M^+^, 100). Anal. calc. for C_26_H_29_N_5_O_2_S: C, 65.66%; H, 6.15%; N, 14.73%; O, 6.73%; S, 6.74%; found: C, 65.66%; H, 6.2%; N, 14.7%; O, 6.7%; S, 6.7%.


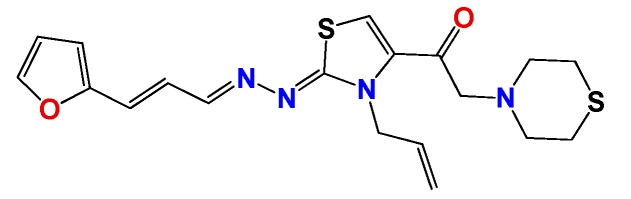


*1-((Z)-3-allyl-2-((E)-((E)-3-(furan-2-yl)allylidene)hydrazono)-2,3-dihydrothiazol-4-yl)-2-thiomorpholinoethanone* (**GATk2**): Yield: 65% dark oil, ^1^H NMR (400 MHz, CDCl_3_) δ: 8.08 (d, *J* = 10 Hz, 1H), 7.70 (s, 1H), 7.45 (bs, 1H), 6.92 (dd, *J* = 16, 10.1 Hz, 1H), 6.66 (d, *J* = 16 Hz, 1H), 6.44 (bs, 2H), 5.95 (m, 1H), 5.13 (m, 2H), 4.93 (m, 2H), 4.28 (bs, 2H), 3.18 (bs, 4H), 3.00 (bs, 4H); ^13^C NMR (100 MHz, CDCl_3_) δ: 170, 169, 149, 146, 145, 134, 130, 130, 125, 118, 112, 112, 110, 62, 51, 48, 40; MS (EI) *m*/*z* (abundance, %): 401.12 (M^+^, 100). Anal. calc. for C_19_H_22_N_4_O_2_S_2_ C: 56.69%; H, 5.51%; N, 13.92%; O, 7.95%; S, 15.93%; found: C, 56.7%; H, 5.5%; N, 13.9%; O, 8.0%; S, 16%.


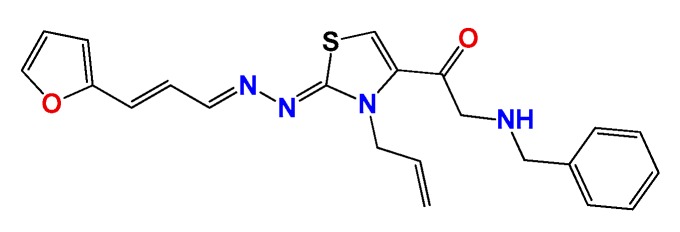


*1-((Z)-3-allyl-2-((E)-((E)-3-(furan-2-yl)allylidene)hydrazono)-2,3-dihydrothiazol-4-yl)-2-(benzylamino)ethanone* (**GATk4**): Yield: 60% dark oil, ^1^H NMR (400 MHz, CDCl_3_) δ: 7.75 (d, *J* = 6 Hz, 1H), 7.69 (s, 1H), 7.50 (d, *J* = 16 Hz, 1H), 7.20–7.35 (m, 5H), 6.95–6.85 (m, 4H), 5.95 (m, 1H), 5.13 (m, 2H), 4.93 (m, 2H), 4.20 (bs, 2H), 3.76 (m, 2H); ^13^C NMR (100 MHz, CDCl_3_) δ: 196, 168, 151, 143, 140, 132, 128, 127, 117, 111, 113, 54, 46; MS (EI) *m*/*z* (abundance, %): 405.15 (M^+^, 100). Anal. calc. for C_22_H_22_N_4_O_2_S: C, 65.00%; H, 5.46%; N, 13.78%; O, 7.87%; S, 7.89%; found: C, 65.0%; H, 5.5%; N, 14.0%; O, 7.9%; S, 8.0%


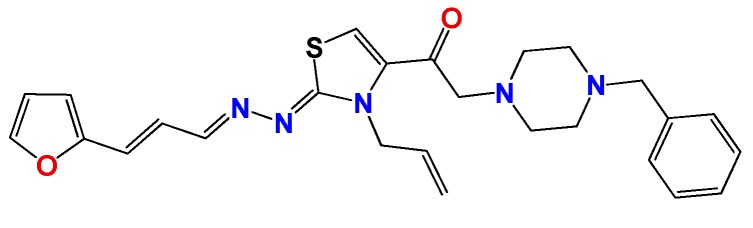


*1-((Z)-3-allyl-2-((E)-((E)-3-(furan-2-yl)allylidene)hydrazono)-2,3-dihydrothiazol-4-yl)-2-(4-benzylpiperazin-1-yl)ethanone* (**GATk5**): Yield: 30% dark oil, ^1^H NMR (400 MHz, CDCl_3_) δ: 8.12 (d, *J* = 10 Hz, 1H), 7.71 (bs, 1H), 7.44 (m, 1H), 7.28 (m, 5H), 6.91 (m, 1H), 6.64 (m, 1H) 6.44 (m, 2H), 5.90 (m, 1H), 5.18 (m, 2H), 5.04 (m, 4H), 4.77 (m, 2H), 3.49 (m, 4H), 2.45 (m, 4H), ^13^C NMR (100 MHz, CDCl_3_) δ: 170, 161, 155, 152, 143, 133, 133, 131, 127, 125, 124, 118, 111, 63, 54, 48; MS (EI) *m*/*z* (abundance, %): 474.20 (M^+^, 100). Anal. calc. for C_26_H_29_N_5_O_2_S: C, 65.66%; H, 6.15%; N, 14.73%; O, 6.73%; S, 6.74%; found: C, 65.7%; H, 6.1%; N, 14.7%; O, 6.7%; S, 6.7%.


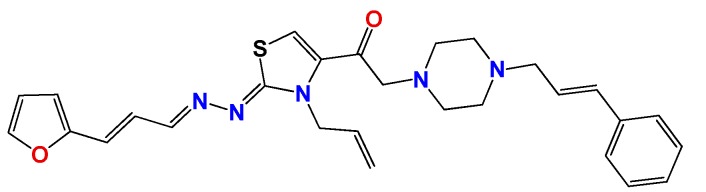


*1-((Z)-3-allyl-2-((E)-((E)-3-(furan-2-yl)allylidene)hydrazono)-2,3-dihydrothiazol-4-yl)-2-(4-cinnamylpiperazin-1-yl)ethanone* (**GATk6**): Yield: 77% dark red solid, m.p. 140–142 °C ^1^H NMR (400 MHz, CDCl_3_) δ: 8.08 (d, *J* = 9.9 Hz, 1H), 7.65–7.16 (m, 7H), 6.94 (dd, *J* = 15.8,9.9 Hz, 1H), 6.75–6.37 (m, 4H), 6.30 (d, *J* = 15.8 Hz, 1H), 5.94 (dd, *J* = 17, 10.4 Hz, 1H), 5.31–5.04 (m, 2H), 4.94 (d, *J* = 5.2 Hz, 2H), 3.81–3.67 (m, 1H), 3.61–3.37 (m, 2H), 3.26 (d, *J* = 6.7 Hz, 2H), 6.65 (bs, 7H), ^13^C NMR (100 MHz, CDCl_3_) δ: 187, 167, 155, 153, 143, 142, 133, 127, 127, 127, 125, 125, 125, 125, 125, 125, 116, 110, 63, 60, 53, 53, 49; MS (EI) *m*/*z* (abundance, %): 500.22 (M^+^, 100). Anal. calc. for C_28_H_31_N_5_O_2_S: C, 67.04%; H, 6.23%; N, 13.96%; O, 6.38%; S, 6.39%; found: C, 67.1%; H, 6.2%; N, 14.0%; O, 6.4%; S, 6.4%.


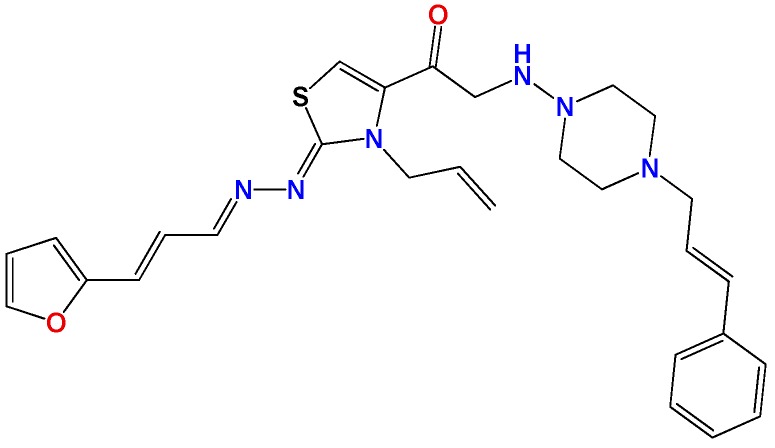


*1-((Z)-3-allyl-2-((E)-((E)-3-(furan-2-yl)allylidene)hydrazono)-2,3-dihydrothiazol-4-yl)-2-((4-cinnamylpiperazin-1-yl)amino)ethanone* (**GAT0613**): Yield: 70% dark red solid, m.p. 150–153 °C ^1^H NMR (400 MHz, CDCl_3_) δ: 8.09 (d, *J* = 10 Hz, 1H), 7.42 (m, 1H), 7.24 (m, 1H), 6.96 (dd, *J* = 16, 10.0 Hz, 1H), 6.62 (m, 2H), 6.42 (m, 2H), 6.28 (m, 1H), 6.21 (m, 5H), 5.95 (m, 1H), 5.16 (m, 2H), 4.93 (m, 2H), 3.43 (bs, 4H), 3.29 (bs, 2H), 2.74 (bs, 4H), ^13^C NMR (100 MHz, CDCl_3_) δ: 167, 163, 160, 153, 143, 142, 135, 128, 127, 126, 125, 125, 125, 117, 116, 116, 110, 110, 60, 51, 49, 48; MS (EI) *m*/*z* (abundance, %): 515.23 (M^+^, 100). Anal. calc. for C_28_H_32_N_6_O_2_S: C, 65.09%; H, 6.24%; N, 16.27%; O, 6.19%; S, 6.21%; found: C, 65.1%; H, 6.2%; N, 16.3%; O, 6.2%; S, 6.2%.

#### 3.1.6. General Synthetic Procedure for Compounds **GAT1113**, **GAT0413**, **GAT0913b** and **GAT1912**

A mixture of the corresponding ketone or aldehyde (1.0 eq.) with the corresponding hydrazine (1.5 eq.) on dry ethanol and catalytic amount of *p*-toluenesulfonic acid (1 mL per 100 mg of reagent) was heated at 50 °C under a calcium chloride chamber until the disappearance of the reagents for 4–10 h (checked by TLC, SiO_2_, petroleum ether:EtOAc 50:50). After that, the mixture was vaporized in vacuum and purified by column chromatography SiO_2_, petroleum ether:EtOAc (0–70% of EtOAc increasing gradually).


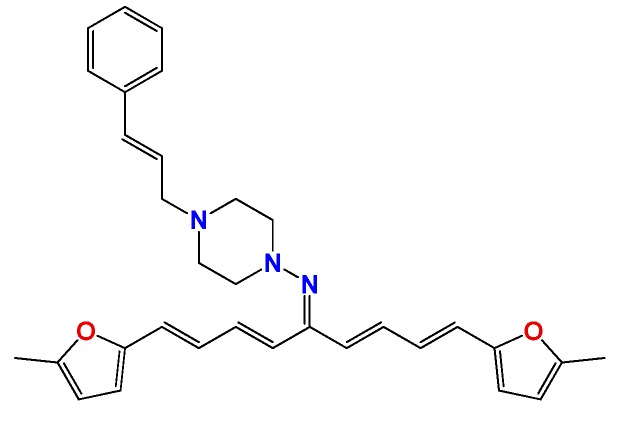


*N-((1E,3E,6E,8E)-1,9-bis(5-methylfuran-2-yl)nona-1,3,6,8-tetraen-5-ylidene)-4-cinnamylpiperazin-1-amine* (**GAT1113**): Yield: 20% dark brown solid, m.p. 148–150 °C ^1^H NMR (400 MHz, CDCl_3_) δ: 7.4–7.2 (m, 5H), 6.97 (dd, *J* = 17, 10.1 Hz, 1H), 6.8–6.7 (m, 4H), 6.55 (d, *J* = 17 Hz, 1H), 6.48–6.40 (m, 2H), 6.28 (m, 2H), 6.23 (d, *J* = 4 Hz, 1H), 6.04 (dd, *J* = 12, 4 Hz, 2H), 3.25 (d, *J* = 8 Hz, 2H), 2.99 (bs, 4H), 2.70 (bs, 4H), 2.39–2.35 (m, 6H). ^13^C NMR (100 MHz, CDCl_3_) δ: 157.6, 155.6, 152.4, 137.8, 136.2, 128.5, 127, 123, 117, 109, 60, 53, 14; MS (EI) *m*/*z* (abundance, %): 492.27 (M^+^, 100). Anal. calc. for C_32_H_35_N_3_O_2_: C, 77.86%; H, 7.15%; N, 8.51%; O, 6.48%; found: C, 78.0%; H, 7.1%; N, 8.5%; O, 6.5%.


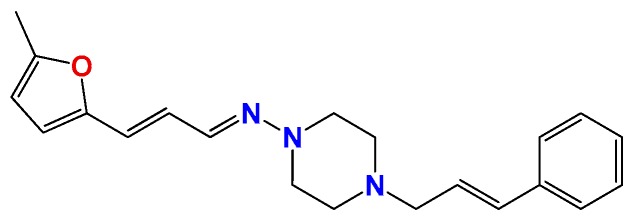


*(E)-4-cinnamyl-N-((E)-3-(5-methylfuran-2-yl)allylidene)piperazin-1-amine* (**GAT0413**): Yield: 69% dark red oil, ^1^H NMR (400 MHz, CDCl_3_) δ: 7.42–7.26 (m, 8H), 6.78 (dd, *J* = 17, 10.1 Hz, 1H), 6.59 (d, *J* = 17 Hz, 1H), 6.41 (d, *J* = 17 Hz, 1H), 6.32 (m, 2H), 6.23 (d, *J* = 4 Hz, 1H), 6.01 (d, *J* = 4 Hz, 1H), 3.22 (m, 6H), 2.69 (m, 4H), 2.32 (s, 3H) ^13^C NMR (100 MHz, CDCl_3_) δ: 157, 152, 136, 132, 128, 127, 125, 117, 109, 60, 53, 14; MS (EI) *m*/*z* (abundance, %): 334.44 (M^+^, 100). Anal. calc. for C_21_H_25_N_3_O: C, 75.19%; H, 7.51%; N, 12.53%; O, 4.77%; found: C, 75.2%; H, 7.5%; N, 12.5%; O, 4.8%.


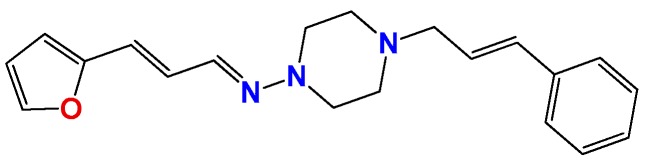


*(E)-4-cinnamyl-N-((E)-3-(furan-2-yl)allylidene)piperazin-1-amine* (**GAT0913b**): Yield: 79% orange solid, m.p. 135–137 °C, ^1^H NMR (400 MHz, CDCl_3_) δ: 7.42–7.26 (m, 7H), 6.86 (dd, *J* = 17, 10.1 Hz, 1H), 6.59–6.29 (m, 5H), 3.21 (bs, 6H), 2.70 (bs, 4H) ^13^C NMR (100 MHz, CDCl_3_) δ: 151, 143, 136, 128, 127, 125, 113, 59.5, 53; MS (EI) *m*/*z* (abundance, %): 334.44 (M^+^, 100). Anal. calc. for C_20_H_23_N_3_O: C, 74.74%; H, 7.21%; N, 13.07%; O, 4.98%; found: C, 74.7%; H, 7.2%; N, 13.1%; O, 4.9%.


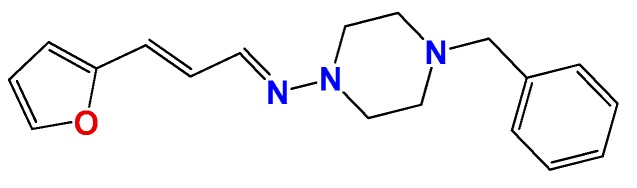


*(E)-4-benzyl-N-((E)-3-(furan-2-yl)allylidene)piperazin-1-amine* (**GAT1912**): Yield: 98% yellow solid, m.p. 145–147 °C, ^1^H NMR (400 MHz, CDCl_3_) δ: 7.40–7.28 (m, 7H), 6.87 (dd, *J* = 17, 10.1 Hz, 1H), 6.47 (d, *J* = 17 Hz, 1H), 6.41 (dd, *J* = 4, 1.7 Hz, 1H), 6.35 (d, *J* = 4 Hz, 1H), 5.59 (s, 2H), 3.18 (m, 4H), 2.65 (m, 4H) ^13^C NMR (100 MHz, CDCl_3_) δ: 151, 143, 138, 132, 128, 127, 125, 113, 114, 64, 59, 52; MS (EI) *m*/*z* (abundance, %): 334.44 (M^+^, 100). Anal. calc. for C_18_H_21_N_3_O: C, 73.19%; H, 7.17%; N, 14.23%; O, 5.42%; found: C, 73.2%; H, 7.2%; N, 14.2%; O, 5.0%.

#### 3.1.7. Synthetic Procedure for Compound (3*E*,5*E*)-3,5-Bis((*E*)-3-(furan-2-yl)allylidene)-1-methylpiperidin-4-one (**Pg150**) [12]


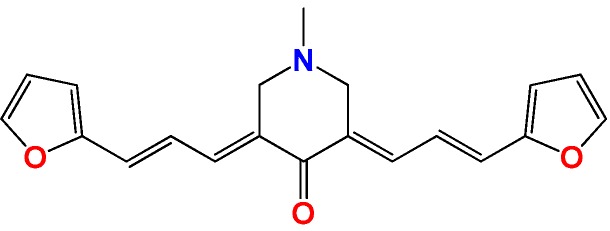


A mixture of the 1-methylpiperidin-4-one (1.0 eq.) and the corresponding aldehyde (2.0 eq.) was dissolved in 1.0 mL of water and 10 mL of ethanol in a 50-mL flask equipped with a magnetic stirrer. Then, NaOH (4.0 eq.) was added, and the reaction mixture was stirred for 24 h at room temperature. The reaction was monitored using TLC with silica as the stationary phase and a mixture of 7:3 hexane/ethyl acetate as the mobile phase. The precipitated solid was filtered under vacuum, washed with water and crystallized from ethanol. Yield = 98% yellow solid, m.p. = 141–144 °C, ^1^H NMR (400 MHz, CDCl_3_) δ: 7.46 (d, *J* = 1.6, 2H), 7.42 (d, *J* = 11.0 Hz, 2H), 6.87 (d, *J* = 11.1 Hz, 2H), 6.75 (d, *J* = 11.1 Hz, 2H), 6.55 (d, *J* = 1.6 Hz, 2H), 6.52 (d, *J* = 11.0 Hz, 2H), 6.47 (d, *J* = 1.8 Hz, 2H), 3.37 (bs, 4H), 2.26 (s, 3H). ^13^C NMR (100 MHz, CDCl_3_) δ: 189, 153, 143, 128, 125, 113, 100, 57, 45; MS (EI) *m*/*z* (abundance, %): 320.14 (M^+^ 100). Anal. calc. for C_20_H_19_NO_3_: C, 74.75%; H, 5.96%; N, 4.36%; O, 14.94%; found: C, 74.8%; H, 6.0%; N, 4.4%; O, 14.9%.

### 3.2. Anti-Parasitic Test In Vitro [15,37]

A culture of *L. braziliensis* (MHOM/BR/75/M2904) and *L. infantum* (MHOM/FR/91/LEM2259V) was obtained from the Facultad de Farmacia (Universidad Complutense, Madrid, Spain). The maintenance of the strains, the form of cultivation and the isolation of the shape of the promastigote were performed following the procedures described by Roldos et al. [38]. The promastigotes were grown at 22 °C in Schneider’s *Drosophila* medium supplemented with 20% FBS. The assay was performed using a modification of a previous method. Promastigotes (2 × 10^6^ parasites/well) were cultivated in 96-well plastic plates. Compounds were dissolved in dimethylsulfoxide (DMSO). Different dilutions of the compounds with a final volume up to 200 µL were added. After 48 h at 26 °C, 20 µL of a 2 mM resazurin solution were added, and the oxidation-reduction was quantified at 570 and 600 nm. The solution of resazurin was prepared at 2.5 mM in phosphate buffered solution (PBS), pH 7.4, and filtered through 0.22-µm membranes prior to use. All tests were carried out in triplicate. Resazurin sodium salt was obtained from Sigma-Aldrich (St. Louis, MO, USA) and stored at 4 °C protected from light. The efficacy of each compound was estimated by calculating the IC_50_ values. Each antiproliferative experiment was done in duplicate, and each concentration was tested in triplicate.

For the in vitro anti-*T. cruzi* activity, we used epimastigotes of the Tulahuen 2 strain (genotype TcVI) grown in an axenic milieu (BHI-Tryptose). Cells from a 5–7-day-old culture were inoculated in fresh culture milieu to give an initial concentration of 1.00 × 10^6^ cells/mL. The absorbance at 600 nm of the cells in culture was measured every day. At Day 5, the milieu was inoculated with different quantities of the compounds from a stock solution in DMSO (DMSO concentration in the culture milieu never exceeded 0.4%). The control was made in the presence of 0.4% DMSO and in the absence of compounds. Each concentration of compound was evaluated in duplicate. At Day 5, the absorbance of the culture was measured and related to the control. The IC_50_ value was taken as the concentration of drug needed to reduce the absorbance ratio to 50%. Each antiproliferative experiment was done in duplicate, and each concentration was tested in triplicate.

### 3.3. Nonspecific In Vitro Cytotoxicity of Mammalian Cells [15,38]

J774.1 murine macrophage cells (ATCC, USA) were grown in DMEM culture milieu containing 4 mM L-glutamine and supplemented with 10% FCS. The cells were seeded in a 96-well plate (5.00 × 10^4^ cells in 200 µL culture medium) and incubated at 37 °C in a 5% CO_2_ atmosphere for 48 h, to allow cell adhesion prior to drug testing. Afterwards, cells were exposed for 48 h to the compounds (25–400 μM) or the vehicle for control (0.4% DMSO), and additional controls (cells in medium) were used in each test. Cell viability was then assessed by measuring the mitochondria-dependent reduction of MTT (3-(4,5-dimethylthiazol-2-yl)-2,5-diphenyltetrazolium bromide) to formazan. For this purpose, MTT in sterile PBS (0.2% glucose), pH 7.4, was added to the macrophages to achieve a final concentration of 0.1 mg/mL, and the cells were incubated at 37 °C for 3 h. After removing the medium, formazan crystals were dissolved in 180 μL of DMSO and 20 μL of MTT buffer (0.1 M glycine, 0.1 M NaCl, 0.5 mM EDTA, pH 10.5), and the absorbance at 560 nm was measured. The IC_50_ was defined as the drug concentration at which 50% of the cells were viable, relative to the control (no drug added), and was determined by analysis using OriginLab8.5^®^ sigmoidal regression (% of viable cells vs. the logarithm of the compound concentration). Tests were performed in triplicate.

NCTC-Clone 929 were grown in minimal essential medium (Sigma) supplemented with 10% heat-inactivated FBS, penicillin G (100 U/mL) and streptomycin (100 µg/mL). Cell cultures were maintained at 37 °C in a humidified 5% CO_2_ atmosphere. The procedure for cell viability measurement was evaluated with resazurin by a colorimetric method. The cells were plated in 96 microtiter plates at 3.00 × 10^4^ cells/well in 100 µL growth medium. The cells were grown overnight at 37 °C, 5% CO_2_. Thereafter, the medium was removed, and the compounds were added to 200 µL of medium for 24 h. After incubation, 20 µL of a 2 mM resazurin solution were added to each well. The plates were incubated for 3 h to allow optimal oxidation-reduction. The reduction of resazurin was determined by dual wavelength absorbance measurement at 490 and 595 nm. The background was subtracted. Each concentration was assayed three times. Medium and drug controls were used in each test as blanks.

### 3.4. Inhibition of Triosephosphate Isomerase [12]

Expression and purification of proteins: *Tc*TIM, *Lm*TIM and *Hs*TIM were expressed in *Escherichia coli* and purified as described in the literature [39]. After purification, the enzyme, dissolved in 100 mM triethanolamine, 10 mM EDTA and 1 mM dithiothreitol (pH 8), was precipitated with ammonium sulfate (75% saturation) and stored at 4 °C. Before use, extensive dialysis against 100 mM triethanolamine/10 mM EDTA (pH 7.4) was performed. Protein concentration was determined by absorbance at 280 nm (ε = 36,440 M^−1^cm^−1^) *Tc*TIM and *Lm*TIM (ε = 33,460 M^−1^cm^−1^) for *Hs*TIM.

Enzymatic activity assays: Enzymatic activity was determined following the conversion of glyceraldehyde 3-phosphate into dihydroxyacetone phosphate. The decrease in absorbance at 340 nm was followed in a multi-cell Cary spectrophotometer at 25 °C. The reaction mixture (1 mL, pH 7.4) contained 100 mM triethanolamine, 10 mM EDTA, 0.2 mM NADH, 1 mM glyceraldehyde 3-phosphate and 0.9 units of α-glycerol phosphate dehydrogenase. The reaction was initiated by the addition of 5 ng/mL of the *Tc*TIM.

For the inhibition studies, *Tc*TIM, *Lm*TIM and *Hs*TIM were incubated at a concentration of 5 mg/mL in a buffer containing 100 mM triethanolamine, 10 mM EDTA, pH 7.4 and 10% of DMSO at 36 °C. The mixture also contained the compounds at the indicated concentrations. Compounds were dissolved in DMSO. After 2 h, 10 µL was withdrawn and added to a final volume of 100 µL of the reaction mixture for the activity assay. The inhibition assay was performed in a 96-well microplate Varioskan spectrophotometer. None of the molecules tested here affected the activity of α-glycerol phosphate dehydrogenase, the enzyme used for trapping the product. The IC_50_ value was taken as the concentration of drug needed to reduce the enzymatic activity to 50%. The experiments were performed in triplicate.

### 3.5. Inhibition of T. cruzi Cruzipain [12]

Cruzipain was purified to homogeneity from epimastigotes of the Tulahuen 2 strain by ConA–Sepharose affinity chromatography, as previously described [40]. Cruzipain (2.5 μM ε = 58,285 M^−1^cm^−1^) was incubated in 50 mM acetate buffer pH 5.5 with 5 mM DTT, and 100 μM inhibitor were added, shaking the solution for 15 min at 27 °C. The derivatives were added as solutions in DMSO, and the controls contained the same solvent concentration. The concentration of DMSO never exceeded 1% in the reaction medium. L-leucylam-ido(4-guanidino)butane (E-64) was used as a positive control of inhibition. Then, the fluorogenic substrate Z-Phe-Arg 7-amido-4-methylcoumarin hydrochloride (Z-Phe-Arg-AMC) at 10 μM was added, and the fluorescence was measured during 10 min at intervals of 3 s (excitation at 380 nm and emission at 460 nm) using a Varioskan Flash Spectrophotometer. From the slope of the negative control, we calculated the total (100%) enzyme activity, while the slopes obtained in the presence of the compounds yielded the percentage of remaining enzyme activity. The percentage of enzyme inhibition was determined as 100% of remaining enzyme activity. The experiments were done in duplicate. 

### 3.6. Nucleophile Challenge [41]

A mixture of cysteine (5.0 eq.), the corresponding compound (1 eq.) in a buffer containing 100 mM triethanolamine, 10 mM EDTA, pH 7.4 and 10% of DMSO was incubated at 36 °C for 2 h. The reaction was checked by TLC and SiO_2_ (with different combinations of petroleum ether:EtOAc) every 15 min, and it was revealed with a mixture of anisaldehyde:EtOH:H_2_SO_4_, UV, I_2_ vapors and Brady reagent.

### 3.7. Adapting the Zebrafish Embryo Toxicity Test (FET) to Lipophilic Drugs

Fish maintenance and embryo production: Zebrafish lines were kept under controlled conditions, in an automated ZebTec (Tecniplast, Milan, Italy) stand-alone system at 28 °C, 500 μS/cm^2^ conductivity, pH 7.5, and fed with dry and live food (*Artemia salina*) three times a day, following accepted protocols and under the approval of the local and national ethical committees, Comisión Honoraria de Experimentación Animal—Universidad de la República (CHEA-UdelaR), and Comisión Nacional de Experimentación Animal (CNEA). The SAT (Sanger AB Tübingen) wild-type line used for most experiments was obtained from Zebrafish International Resource Center (http://zebrafish.org) (Eugene, OR, USA). Embryos obtained from natural crossings were cultured at 28.5 °C in system water and methylene blue (1 ppm) as a fungistatic. 1-Phenyl-2-thiourea (PTU, 0.003%) was added to system water to inhibit melanogenesis in embryos destined for microscopic imaging.

#### 3.7.1. Embryo Toxicity Test [42,43]

For the assay, 6 hpf embryos were dechorionated in agarose-bottom dishes. Only embryos that were clearly undamaged after 60 min of observation were kept and subsequently used. Dechorionated embryos were further manipulated using flame-rounded tip glass Pasteur pipettes. Dechorionated embryos (20 per condition) were then transferred to round-bottom 96-well plates (1 embryo/well) for incubation with drugs. In all cases, the embryos were incubated in system water with 1% DMSO (negative control) and the compound at different doses between 8 and 96 hpf. The treatment solution was changed daily. As a positive control, we used 2.4 mM caffeine (Figure S1 shows the characteristic morphology for this treatment), and the negative control was 1% DMSO in system water. The embryos were placed at 28.5 ± 0.5 °C in a 96-round bottom well plate. At selected time points, namely 24, 48, 72 and 96 hpf, embryotoxicity (= mortality) and morphological characteristics of the embryos were evaluated using a Nikon SMZ800 stereomicroscope. The embryos were evaluated for the presence and morphological development (as appropriate) of somites, tail detachment, eyes, heart beat and blood circulation. All individuals were evaluated at all time points.

#### 3.7.2. Determination of Drug Bioconcentration [33]

Following the same conditions as those of the embryo toxicity test, five embryos were incubated per well in 6-well plates with 5 mL of 25 μM **GAT1033**. Embryos were incubated with and without chorion, starting at 6 hpf. The incubation medium containing the compound was exchanged daily. Every 24 h, 30 embryos having the same conditions were taken to extract the compound for quantitation. The embryos were washed three times with aquarium water and then centrifuged at low speed to pull out the total remaining solvent. The embryos were then resuspended in 1 mL of aquarium water and lysed by sonication (Omni Sonic Ruptor Omni Sonic Ruptor 400 Ultrasonic Homogenizer) on an ice bath with 4-times half-intensity-15-second pulses, resting 5 seconds. The extract was incubated with 1 mL of methanol/chloroform (2:1) for 5 min at 25 °C, followed by five chloroform extractions. The organic phase was dried over anhydrous sodium sulfate and evaporated in vacuo. The crude extract was dissolved in 100 µL acetonitrile (Merck, HPLC grade) for spectrophotometric measurements (Spectrophotometer double beam UV-Vis model Cary 60 Agilent Cary WinUV). The calibration curve and all of the experiments were performed with **GAT1033** under the same conditions. For this, we used the Beer–Lambert–Bouguer Law (BLB law). Optical data for **GAT1033** are: ε = (14.7 ± 0.5) cm^−1^mM^−1^, λ_max_ = 379 nm, emission 530 nm.

#### 3.7.3. Fluorescence Measurements Using Laser Scanning Confocal Microscopy

The 24 hpf zebrafish embryos, with and without chorion, were exposed to **GAT1033** for 18 h. Image acquisition was made with a Leica TCS-SP5 laser scanning confocal microscope (Leica Microsystems GmbH, Wetzlar, Germany) using LASAF v. 2 software (Leica Microsystems, Wetzlar, Germany) and observed using a 405-nm laser excitation line and an emission bandwidth from 428–646 nm under a 20 × water immersion objective. Further image analysis, including quantitation, and processing of figures were made using Fiji (http://fiji.sc/) [44].

## 4. Conclusions

In this work, we introduce a new class of anti-kinetoplastid agents. We evaluated the anti-kinetoplastid effect of forty of these compounds on *Leishmania* spp. Fourteen compounds were active against *Leishmania* spp. (IC_50_ < 25 µM), and seven compounds had multi-anti parasitic activity against the three species of parasites. Furthermore, we demonstrated that two different chemical motifs could be combined to obtain a new multi-anti-parasitic compound (the furylacroleine and cinnamylpiperazine motifs). We also found six compounds suitable for drug development against Leishmaniasis, which have good toxicological and synthetic profiles. **GAT1033**, **EA138, HIT1**, **HIT2**, **GAT1113** and **EA155** can continue the following stages of development (such as the in vivo assay models for Leishmaniasis in mouse). Structural modification of compound **GAT1113** could be useful to find new multi-anti-parasitic compounds (using combinations between active diarylideneketones with a cinnamylpiperazine motif). These compounds have a good selectivity index and low-cost production, an important characteristic in drugs for neglected diseases. The mechanism of action for these compounds in *Leishmania* spp. is not clear; the levels of inhibition for triosephosphate isomerase and cruzipain cannot explain the complete trypanocidal effect. **HIT1** had good levels of inhibition of cruzipain, comparable to reported inhibitors. We also adapted the classic toxicological test for lipophilic compounds in zebrafish, considering their bioconcentration effects.

## Figures and Tables

**Table 1:** (**A**) Trypanocidal activity against *T. cruzi* and *Leishmania* spp. for the thiazolidenehydrazine derivatives. The test was carried out on *T. cruzi* epimastigotes and *L. braziliensis* and *L. infantum* promastigotes. We show only the best trypanocidal compounds; the complete data are in Table S1 of the Supporting Material. The multi-anti-parasitic compounds are shown on a green background; (**B**) Trypanocidal activity against *T. cruzi* and *Leishmania* spp. for the diarylideneketones. The test was carried out on *T. cruzi* epimastigotes and *L. braziliensis* and *L. infantum* promastigotes. We show only the best trypanocidal compounds; the complete data are in Table S1 of the Supporting Material. The multi-anti-parasitic compounds are shown on a green background.

**A**				
**Structure**	**Compound**	**IC_50_ ± SD ^a^ (µM) Epimastigotes *T. cruzi***	**IC_50_ ± SD (µM) Promastigotes *L. braziliensis***	**IC_50_ ± SD (µM) Promastigotes *L. infantum***
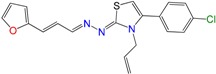	**GAT1033 ^b^**	1.6 ± 0.5 ^c^	7 ± 1	2.0 ± 0.2
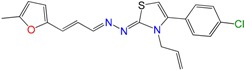	**GAT0113A**	3.0 ± 0.5	10 ± 1	8 ± 2
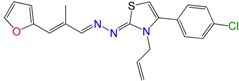	**GAT0513**	0.09 ± 0.02	33 ± 11	58 ± 12
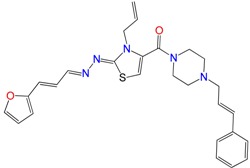	**HIT1**	3.1 ± 0.2 ^d^	12 ± 5	4 ± 1
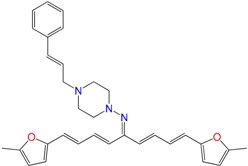	**GAT1113**	1.6 ± 0.3	16 ± 4	14 ± 2
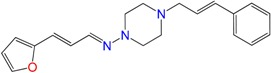	**GAT0913b**	25 ± 8	18 ± 5	21 ± 2
	Glucantime	-	18 ± 2	26 ± 9
	Miltefosine	8 ± 1	-	0.9 ± 0.2
	Benznidazole	7 ± 1	-	-
**B**				
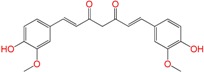	Curcumin	5.6 ± 1 ^e^	-	5.9 ± 0.3 ^b^
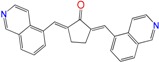	**Comp13 ^f^**	31 ± 2 ^f^	0.9 ± 0.2 ^c^	-
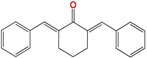	**EA142 ^g^**	5.1 ± 0.3 ^h^	4.2 ± 0.7	9.6 ± 0.9
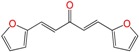	**EA134**	24 ± 2 ^h^	10 ± 6	6 ± 2
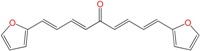	**EA128**	5.0 ± 0.7 ^h^	36 ± 9	31 ± 9
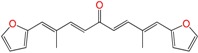	**EA155**	8.2 ± 2.0 ^h^	16 ± 2	6 ± 1
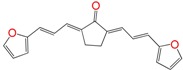	**EA161**	5.4 ± 1.6 ^h^	18 ± 4	16 ± 4
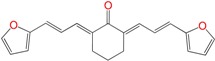	**HIT2**	0.6 ± 0.2 ^h^	7 ± 1	13 ± 7
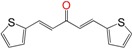	**EA138**	5.0 ± 0.8 ^h^	8 ± 2	4.0 ± 0.5
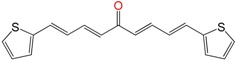	**EA141**	12.6 ± 1.4 ^h^	36 ± 3	19 ± 5
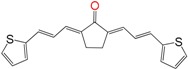	**EA160**	0.04 ± 0.01 ^h^	>100	11 ± 3
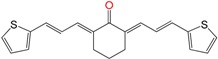	**EA156**	0.6 ± 0.2 ^h^	>100	16 ± 3
	Glucantime	-	18 ± 2	26 ± 9
	Miltefosine	8 ± 3	0.9 ± 0.2	-
	Benznidazole	7 ± 1	-	-

^a^ Standard deviation on the triplicate; ^b^ The compounds codes are exactly the same from the personal codes on the Lab (**GATN°**, **GATkN°**, **GATjmN°**, **EAN°** and **PgN°**); ^c^ Data from [13]; ^d^ data from [15]; ^e^ Data from [21]; ^f^ Data from [22] (the compound code is exactly the same from the reference); ^g^ The compounds codes are exactly the same from the personal codes on the Lab (**GATN°**, **GATkN°**, **GATjmN°**, **EAN°** and **PgN°**); ^h^ Data from [12].

**Table 2:** Nonspecific cytotoxicity for mammalian cells. The test was carried out on NCTC929 fibroblasts and J774.1 murine macrophages. We show the selectivity indexes for *L. braziliensis* (promastigotes), *L. infantum* (promastigotes) and *T. cruzi* (epimastigotes), respectively. The color code highlights the good (green ˃ 25), medium (yellow 25–5) and bad (red < 5) selectivity indexes (SI).

Compound	IC_50_ ± SD (µM) Fibroblast NCTC929	IC_50_ ± SD (µM) Murine Macrophages	SI ^e^ NCTC/ *L. braziliensis*	SI NCTC/ *L. infantum*	SI J774.1/ *T. cruzi*
**GAT1033**	405 ± 10	60 ± 6 ^a^	58	203	37 ^a^
**GAT0113A**	319 ± 16	66 ± 7	32	40	22
**GAT0513**	1443 ± 30	55 ± 5	44	25	611
**HIT1**	346 ± 9	30 ± 5 ^b^	29	87	10
**GAT1113**	165 ± 5	45 ± 5	10	12	28
**GAT0913b**	160 ± 7	25 ± 3	9	8	1
Curcumin	-	10 ± 2 ^d^	2	-	2
**Comp13**	-	21± 5 ^d^	23	-	1
**EA134**	114 ± 2	115 ± 6 ^c^	11	19	5 ^c^
**EA128**	494 ± 25	60 ± 3 ^c^	14	16	12 ^c^
**EA155**	543 ± 15	33 ± 8 ^c^	34	91	4 ^c^
**EA161**	756 ± 17	19 ± 2 ^c^	42	47	4 ^c^
**HIT2**	160 ± 9	10 ± 2 ^c^	23	12	17 ^c^
**EA138**	158 ± 5	38 ±7 ^c^	20	40	8 ^c^
**EA141**	1704 ± 40	10 ± 2 ^c^	47	90	1 ^c^
**EA160**	4909 ± 36	15 ± 1 ^c^	nc	446	375 ^c^
**EA156**	1985 ± 20	20 ± 1 ^c^	nc	124	33 ^c^
Glucantime	-	15 ± 1	1	0.5	-
Miltefosine	-	50 ± 7	-	56	6
Benznidazole	-	400 ± 4	-	-	57

^a^ Data from [13]; ^b^ Data from [15]; ^c^ Data from [12]; ^d^ Data from [21,22]. ^e^ SI is the ratio between IC_50_ mammalian cell and IC_50_ in parasite.

**Table 3:** Inhibition of the enzymatic activity of triosephosphate isomerase and cruzipain.

Compound	Percentage of Inhibition ^a,b^/IC_50_ ± SD (µM) *Tc*TIM	Percentage of Inhibition ^a,b^/IC_50_ ± SD (µM) *Lm*TIM ^c^	Percentage of Inhibition ^a,b^/IC_50_ ± SD (µM) Cruzipain
**GAT1033**	0/-	0/-	0/-
**HIT1**	0/-	0/-	100/4.3 ± 0.4
**EA128**	100/3.0 ± 0.7 ^d^	0/-	48/- ^d^
**EA155**	100/3.3 ± 0.5 ^d^	100/˂25 ^e^	0/- ^d^
**HIT2**	100/0.086 ± 0.007 ^d^	100/˂25 ^e^	80/37.0 ± 1.1 ^d^
**EA138**	0/- ^d^	100/˂25 ^e^	50/- ^d^

^a^ Percentage of enzymatic inhibition at 100 µM; ^b^ The experiments were done in triplicate; ^c^ Triosephosphate isomerase from *Leishmania mexicana*; ^d^ Data from [17]; ^e^ The IC_50_ values is between 12.5 and 25 µM.

## Supplementary Materials

Supplementary materials are available online.
