# Supplementary material for: Multi-Anti-Parasitic Activity of Arylidene Ketones and Thiazolidene Hydrazines against Trypanosoma cruzi and Leishmania spp
Source: Molecules. 2017 May 7;22(5):709. doi: 10.3390/molecules22050709 (PMC6154605; doi:10.3390/molecules22050709)
Supplement: Supplementary file 1 [file molecules-22-00709-s001.pdf]

Multi-anti-parasitic activity of arylideneketones and thiazolidenehydrazines against *Trypanosoma cruzi* and *Leishmania spp*.

Guzmán Álvarez[1*], Cintya Perdomo[1], Cathia Coronel[2], Elena Aguilera[3], Javier Varela[3], Gonzalo Aparicio[4,5], Flavio R. Zolessi[4,5], Nallely Cabrera[6], Ruy Pérez-Montfort[6], Celeste Vega[2], Miriam Rolón[2], Antonieta Rojas de Arias[2] Hugo Cerecetto[3], Mercedes González[3].

[1] Laboratorio de Moléculas Bioactivas, CENUR Litoral Norte, Universidad de la República, Ruta 3 (km 363), Paysandú, C.P. 60000, Uruguay.

[2] Centro para el Desarrollo de la Investigación Científica (CEDIC/FMB/Diaz Gill Medicina Laboratorial), Asunción, Paraguay.

[3] Grupo de Química Medicinal-Laboratorio de Química Orgánica, Facultad de Ciencias, Universidad de la República, Montevideo, Uruguay.

[4] Sección Biología Celular, Facultad de Ciencias, Universidad de la República, Uruguay.

[5] Institut Pasteur de Montevideo, Uruguay.

[6] Departamento de Bioquímica y Biología Estructural, Instituto de Fisiología Celular, Universidad Nacional Autónoma de México, CD México, México.

* Author to whom correspondence should be addressed (GA); E-Mails: guzmanalvarezlqo@gmail.com; Tel.: +59899274984.

Table 1S. Total *in vitro* data on epimastigotes *T. cruzi*, promastigotes *L. braziliensis* and promastigotes *L. infantum*.

**Structure**	**Compound**	**IC50 ± %DS (µM) epimastigotes *T. cruzi***	**IC50 ± %DS (µM) promastigotes *L. braziliensis***	**IC50± %DS (µM) promastigotes *L. infantum***
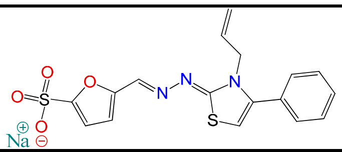	**GAT0812**	>25	>100	>100
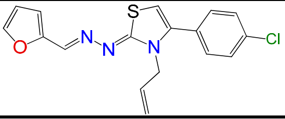	**GAT03311**	>25	>100	22 ± 3
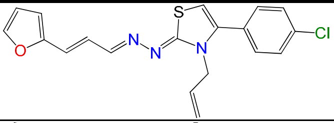	**GAT1033**	1.6 ± 0.5	7 ± 1	2.0 ± 0.2
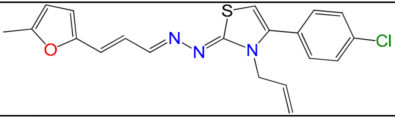	**GAT0113A**	3.0 ± 0.5	10 ± 1	8 ± 2
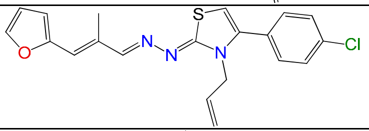	**GAT0513**	0.09 ± 0.02	33 ± 11	58 ± 12
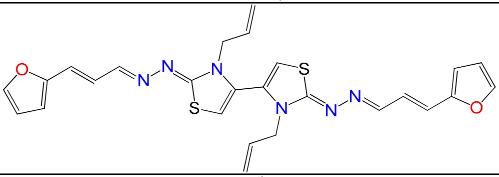	**GAT1082**	3.5 ± 0.2	> 100	> 100
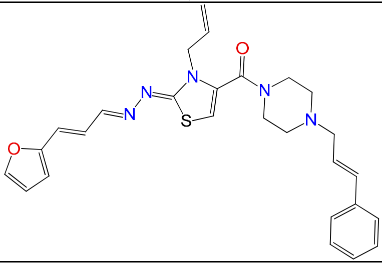	**HIT1**	3.1 ± 0.2	12 ± 5	4 ± 1
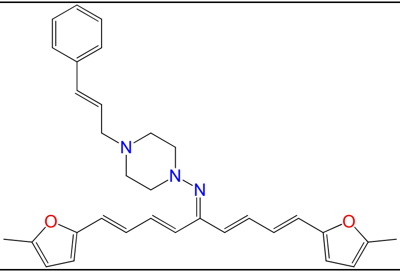	**GAT1113**	1.6 ± 0.3	16 ± 4	14 ± 2
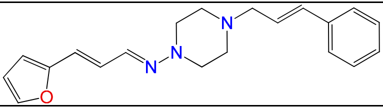	**GAT0913b**	>25	18 ± 5	21 ± 2
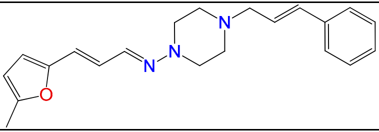	**GAT0413**	12 ± 2	>25	>25
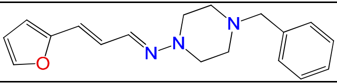	**GAT1912**	>25	> 100	> 100

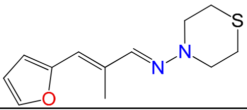	**GAT1013**	>25	>100	>100
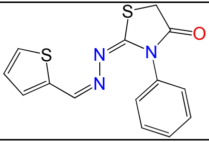	**GAT1075**	>25	>100	>100
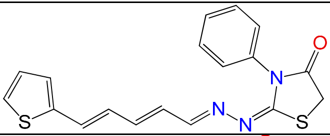	**GAT10117**	>25	>100	>100
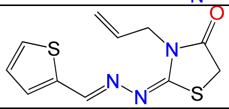	**GAT1049**	>25	>100	>100
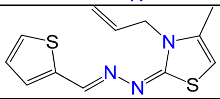	**GAT1050**	>25	>100	72 ± 15
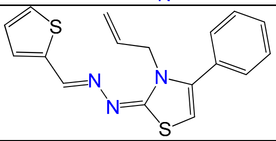	**GAT1048**	>25	90 ± 20	51 ± 11
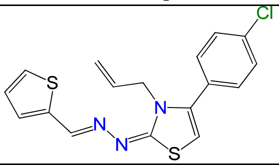	**GAT0921**	>25	>100	33 ± 3
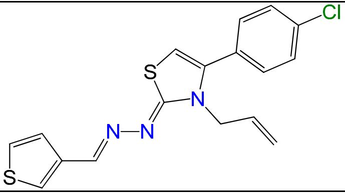	**GATR22**	15 ± 3	23 ± 9	32 ± 12
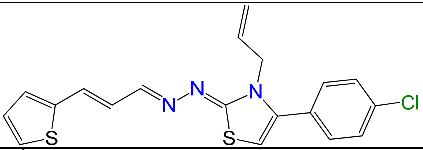	**GAT02111**	>25	>100	39 ± 11
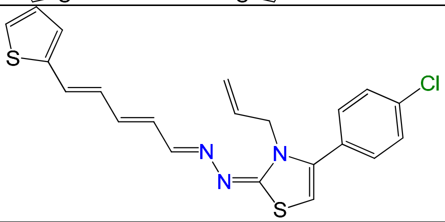	**GAT1066**	13 ± 2	>100	>100
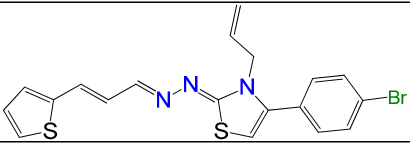	**GAT02211**	>25	>100	>100
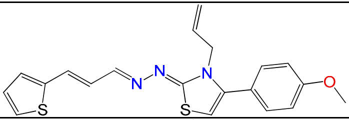	**GAT01811**	31 ± 10	>100	34 ± 12
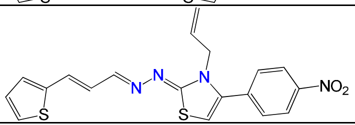	**GAT01911**	>50	>100	>100

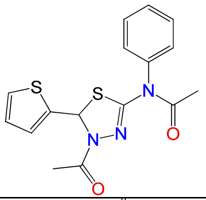	**GAT1057**	>25	>100	>100
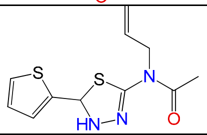	**GAT1058**	>25	>100	>100
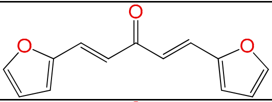	**EA134**	24 ± 2	10 ± 6	6 ± 2
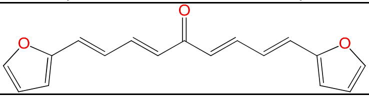	**EA128**	5.0 ± 0.7	36 ± 9	31 ± 9
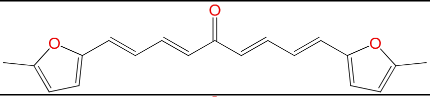	**GAT0813**	9.4 ± 1.4		
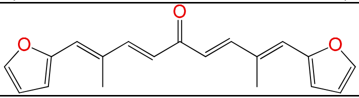	**EA155**	8.2 ± 2.0	16 ± 2	6 ± 1
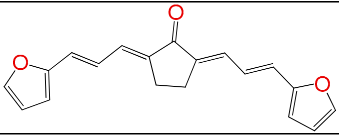	**EA161**	5.4 ± 1.6	18 ± 4	16 ± 4
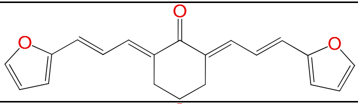	**HIT2**	0.6 ± 0.2	7 ± 1	13 ± 7
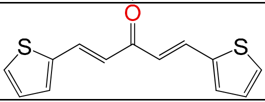	**EA138**	5.0 ± 0.8	8 ± 2	4.0 ± 0.5
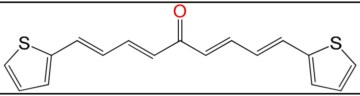	**EA141**	12.6 ± 1.4	36 ± 3	19 ± 5
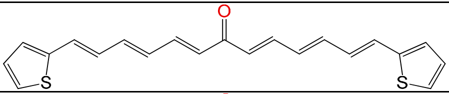	**EA152**	>25	>100	30 ± 8
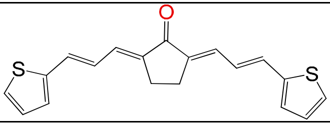	**EA160**	0.04 ± 0.01	>100	11±3
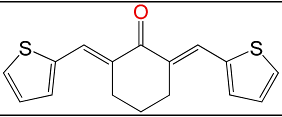	**EA153**	3.6 ± 0.9	>100	>100
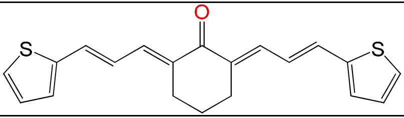	**EA156**	0.6 ± 0.2	>100	16±3
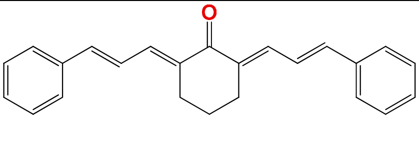	**EA143**	14 ± 3	>25	>25
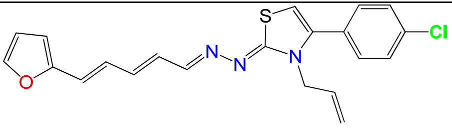	**GATA2**	10 ± 2	nd*	nd

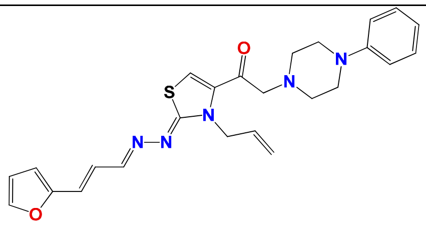	**GATk1**	>25	nd	Nd
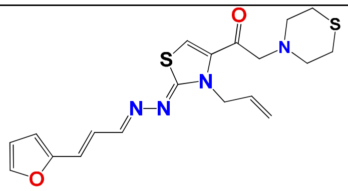	**GATk2**	8 ± 1	nd	Nd
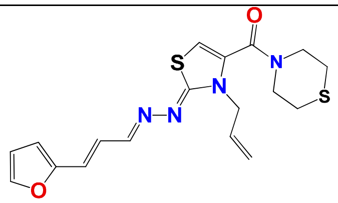	**GATjm18**	2.7 ± 0.5	nd	Nd
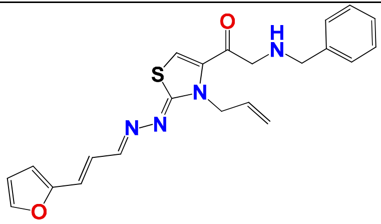	**GATk4**	>25	nd	Nd
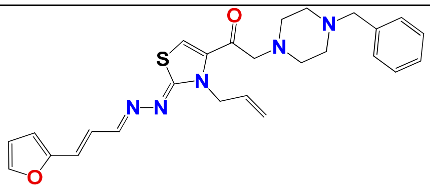	**GATk5**	>25	nd	Nd
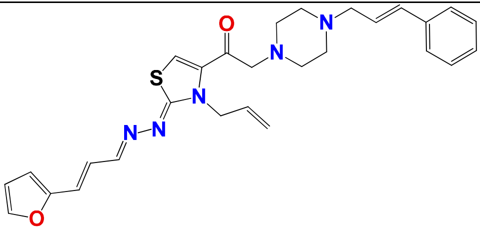	**GATk6**	1.2 ± 0.2	>25	>25
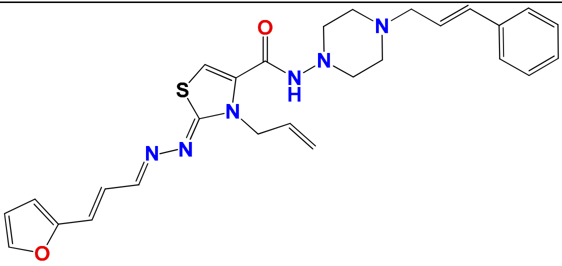	**GAT1613**	20 ± 2	nd	Nd

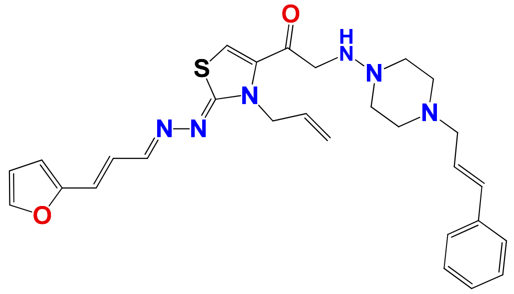	**GAT0613**	11 ± 4	25	>25
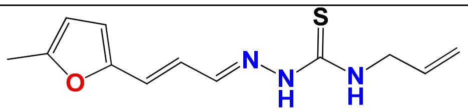	**GAT2512**	5 ± 1	>25	23
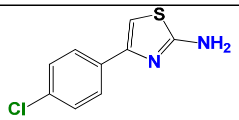	**GAT2012**	>25	nd	nd
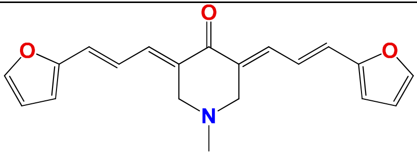	**Pg 150**	>25	>25	>25
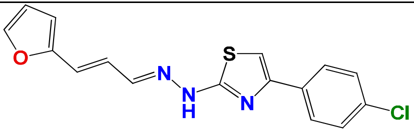	**GAT2212B**	1.2 ± 0.3	>25	25
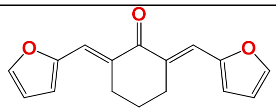	**EA154**	7 ± 2	>25	>25
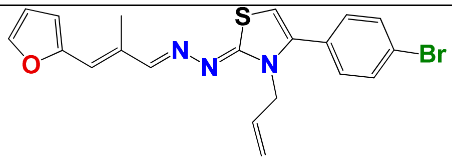	**GAT2015**	nd	>25	>25
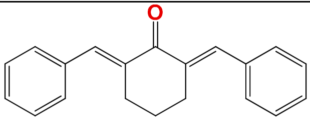	**EA142**	5.1 ± 0.3	4.2 ± 0.7	9.6 ± 0.9
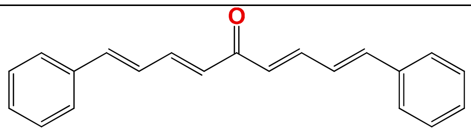	**EA139**	11 ± 1	>25	>25

*nd not determined

Table 2S. Some calculated properties for the compound utilized in this work. Commercial drugs for Chagas disease. Nifurtimox (A) and Benznidazole (B). Drug in clinical stages, ketoconazole (C) and two candidates in preclinical stage (**GAT1033** and **HIT1**).

	Structures	cLopP*	Solubility*
A	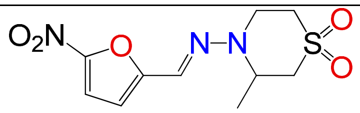	-0.25	-3.0
B	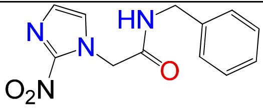	0.66	-2.8
C	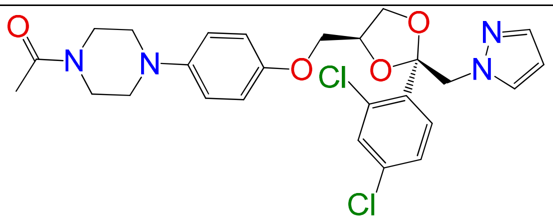	3.0	-3.3
D	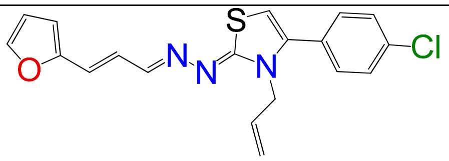	6.0	-5.9
E	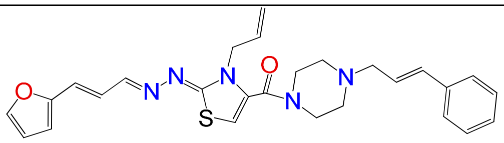	5.3	-4.8

*theoretically calculated by http://molinspiration.com/cgi-bin/properties plataform.

Table 3S. LD_{50} of the assayed compounds.

Compound	LD_{50} (µM) with chorion	LD_{50} (µM) without chorion
**Caffeine**	nd	3000 ± 100
**Benznidazole**	>1000	>1000
**Nifurtimox**	>300	314 ± 4
**Ketoconazole**	>25	12 ± 3
**GAT1033**	>100*	27± 1
**HIT1**	>100*	61± 2

* The chorions were dark, because the aggregation of the compound in it. It was not possible visualize the toxic effect.

Figure 1S. Characteristic morphology of caffeine treatment on zebrafish embryo at 2.4 mM. The tail of the zebrafish embryo treated with caffeine is like a pig tail.

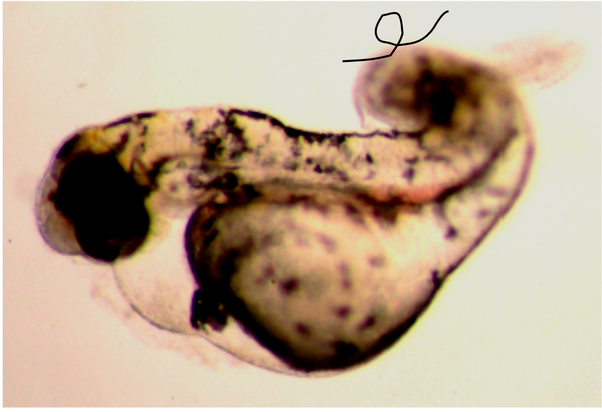

Table 4S. Absorbance data and concentration of the **GAT1033** in zebrafish embryos
.

	O.D.$_{\lambda=396\ nm}$ 24 h		conc. (μM)	O.D.$_{\lambda=396\ nm}$ 48 h		conc. (μM)	O.D.$_{\lambda=396\ nm}$ 72 h		conc. (μM)	Volume of measurement (μL)
control	0.002	0.003	0.0	0.007	0.006	0.0	0.004	0.015	0.0	100
With chorion	0.004	0.006	0.3	0.072	0.058	4.8	0.028	0.024	1.8	100
Without chorion	0.005		0.4	0.109		7.3	0.121		8.1	100
